# UNISOM: Unified Somatic Calling and Machine Learning-based Classification Enhance the Discovery of CHIP

**DOI:** 10.1093/gpbjnl/qzaf040

**Published:** 2025-04-29

**Authors:** Shulan Tian, Garrett Jenkinson, Alejandro Ferrer, Huihuang Yan, Joel A Morales-Rosado, Kevin L Wang, Terra L Lasho, Benjamin B Yan, Saurabh Baheti, Janet E Olson, Linda B Baughn, Wei Ding, Susan L Slager, Mrinal S Patnaik, Konstantinos N Lazaridis, Eric W Klee

**Affiliations:** Division of Computational Biology, Department of Quantitative Health Sciences, Mayo Clinic, Rochester, MN 55905, USA; Division of Computational Biology, Department of Quantitative Health Sciences, Mayo Clinic, Rochester, MN 55905, USA; Division of Hematology, Department of Internal Medicine, Mayo Clinic, Rochester, MN 55905, USA; Division of Computational Biology, Department of Quantitative Health Sciences, Mayo Clinic, Rochester, MN 55905, USA; Department of Microbiology and Immunology, Vanderbilt University Medical Center, Nashville, TN 37232, USA; Division of Computational Biology, Department of Quantitative Health Sciences, Mayo Clinic, Rochester, MN 55905, USA; Division of Hematology, Department of Internal Medicine, Mayo Clinic, Rochester, MN 55905, USA; Department of Computer Science, Stanford University, Stanford, CA 94305, USA; Division of Computational Biology, Department of Quantitative Health Sciences, Mayo Clinic, Rochester, MN 55905, USA; Division of Epidemiology, Department of Quantitative Health Sciences, Mayo Clinic, Rochester, MN 55905, USA; Division of Hematopathology, Department of Laboratory Medicine and Pathology, Mayo Clinic, Rochester, MN 55905, USA; Division of Hematology, Department of Internal Medicine, Mayo Clinic, Rochester, MN 55905, USA; Division of Computational Biology, Department of Quantitative Health Sciences, Mayo Clinic, Rochester, MN 55905, USA; Division of Hematology, Department of Internal Medicine, Mayo Clinic, Rochester, MN 55905, USA; Division of Gastroenterology and Hepatology, Mayo Clinic, Rochester, MN 55905, USA; Division of Computational Biology, Department of Quantitative Health Sciences, Mayo Clinic, Rochester, MN 55905, USA

**Keywords:** Clonal hematopoiesis of indeterminate potential, Machine learning, Somatic variant calling, Whole-genome sequencing, Whole-exome sequencing

## Abstract

Clonal hematopoiesis (CH) of indeterminate potential (CHIP), driven by somatic mutations in leukemia-associated genes, confers increased risk of hematologic malignancies, cardiovascular disease, and all-cause mortality. In blood of healthy individuals, small CH clones can expand over time to reach 2% variant allele frequency (VAF), the current threshold for CHIP. Nevertheless, reliable detection of low-VAF CHIP mutations is challenging, often relying on deep targeted sequencing. Here, we present UNISOM, a streamlined workflow for enhancing CHIP detection from whole-genome and whole-exome sequencing data that are underpowered, especially for low VAFs. UNISOM utilizes a meta-caller for variant detection, in couple with machine learning models which classify variants into CHIP, germline, and artifact. In whole-exome sequencing data, UNISOM recovered nearly 80% of the CHIP mutations identified via deep targeted sequencing in the same cohort. Applied to whole-genome sequencing data from Mayo Clinic Biobank, it recapitulated the patterns previously established in much larger cohorts, including the most frequently mutated CHIP genes and predominant mutation types and signatures, as well as strong associations of CHIP with age and smoking status. Notably, 30% of the identified CHIP mutations had < 5% VAFs, demonstrating its high sensitivity toward small mutant clones. This workflow is applicable to CHIP screening in population genomic studies. The UNISOM pipeline is freely available at https://github.com/shulanmayo/UNISOM and https://ngdc.cncb.ac.cn/biocode/tool/7816.

## Graphical abstract

**Graphical abstract:**
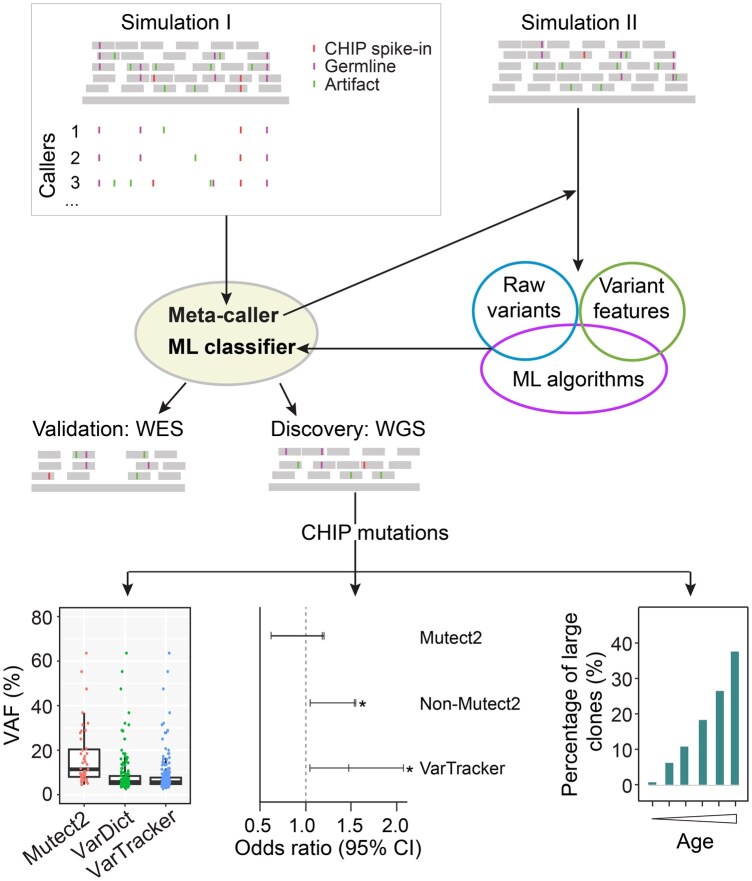

## Introduction

Clonal hematopoiesis (CH) of indeterminate potential (CHIP) is a premalignant state, in which hematopoietic stem cells acquire somatic mutations in leukemia-associated driver genes, leading to clonal overrepresentation in peripheral blood. As currently defined, these somatic mutations have variant allele frequencies (VAFs) of 2% or greater, but the individual does not meet the World Health Organization diagnostic criteria for a hematologic neoplasm [1,2]. Among the candidate CHIP genes [3], *DNMT3A*, *TET2*, and *ASXL1* are most frequently mutated, together harboring over two-thirds of the CHIP mutations identified [4,5]. Therein the cutoff of ≥ 2% VAF for CHIP was chosen due to the limitation of standard next-generation sequencing platforms in detecting smaller clones and the presumed rarity of clinical consequences associated with mutations at lower VAFs [5,6].

Biologically, it has been observed that CHIP prevalence increases markedly with age, from < 2% of individuals under 50 years of age to approximately 10%–20% of those over age 70 [7,8]. Targeted error-corrected sequencing data showed that at least 95% of the healthy adults above 50 years old carry CH [6,9]. Importantly, in general population, the presence of CHIP in the peripheral blood of carriers was associated with an 11-fold increased risk of developing hematologic malignancies [7], and two to four times greater risk of cardiovascular diseases [7,10]. Furthermore, in non-Hodgkin lymphoma [11] and multiple myeloma patients [12] who received autologous stem-cell transplantation, the prevalence of CHIP at the time of therapy is associated with unfavorable overall survival. Notably, individuals with CH of ≥ 1% VAF in leukemia driver genes also had a significantly increased risk of developing acute myeloid leukemia (AML) [6], which corroborated similar findings in larger AML cohorts [13,14].

While CH mutations are nearly universal in healthy individuals over age 50, the vast majority of them have low VAFs with negligible risk of malignant transformation [9]. Overall, large clones are more likely to be associated with the risk of malignant transformation, depending on the mutations, the genes carrying the mutations, and the number of driver mutations. Large clones in *TP53*, *DNMT3A*, *TET2*, and splicing factor genes [13,14], the presence of two or more mutations (*versus* a single one) in *DNMT3A* [13], and the presence of additional driver mutations [14,15], are associated with higher risk of progression to AML.

Clone size is determined by the time of onset and growth rate. *JAK2^V617F^*, *DNMT3A*, and *PPM1D* mutations occurred early in life during childhood, even in utero for the first two [15]. In contrast, *SRSF2^P95H^* and *U2AF1* mutations occurred after 30 years of age and showed rapid expansion [16]. Studies with longitudinal data showed that about 44% of the detected CH clones grew over time [9], which rose to 52% if only considering those with ≥ 1% VAF [17]. The growth rate, which varies markedly among mutations and genes [16,18], is age- and context-dependent. Over 92% of the clones were predicted to grow at fixed rates [16]. However, clones carrying *DNMT3A* and *TP53* mutations had faster expansion early in life, in contrast with those carrying *TET2* and splicing factor gene mutations that grew faster in older individuals [16]. Among these genes, clones in *SRSF2* and *U2AF1* showed faster expansion than those in *DNMT3A* and *TP53* [16]. Further, in the same gene, growth rate also varied by mutations, where *DNMT3A^R882H^* [4,7–9], *TP53* missense mutations [16], and *SRSF2^P95H^* [16] showed faster expansion compared to other mutations.

Several known factors, as well as the so-called unknown-cause effect beyond mutation [16], are associated with clone expansion. For example, a noncoding germline variant rs34002450 in the telomerase reverse transcriptase (*TERT*) gene is associated with increased risk of acquiring CHIP [4,19]. Prior exposure to cancer therapies is also associated with an increased prevalence of CH [20,21]. In non-hematologic cancer, chemoradiation [20] and chemotherapy [21] led to increase in the number of *TP53* mutations and clone expansion. Third, it was estimated that about 5% of the non-synonymous mutations were under positive selection, representing drivers for CH expansion [22]. Finally, the presence of additional driver mutations also contributed to clonal expansion [15]. After the acquiring of *SRSF2* and *CUX1* mutations prior to being diagnosed with AML, clonal expansion was observed in *TP53*, from 3% VAF at baseline to 21% VAF in the third year follow-up [13]. Thus, it is important to differentiate the vast majority of small CH clones that are clinically silent from those that are present in high-risk CHIP genes or show rapid expansion over time.

Accurate screening for CH, including CHIP, requires the detection of low-frequency somatic mutations, which depends heavily on the sequencing platform and depth. Deep targeted sequencing (> 400×) can reach lower limits of 0.5%–1% VAFs [5,21], and even of 0.2% when combined with single-molecule molecular inversion probes (smMIPs, with > 4000× coverage) [23]. Targeted error-corrected sequencing can detect variants with VAFs as low as 0.01% [6]. Whole-exome sequencing (WES) and Whole-genome sequencing (WGS) data from large-scale genomic projects, such as Trans-Omics for Precision Medicine (TOPMed) and UK Biobank, have been repurposed for the discovery of CHIP mutations [4,24]. However, at ∼ 100× depth of coverage, typically seen in WES, the majority of the CHIP mutations with 2%–5% VAFs will not be reliably detected with current somatic calling practice [4], which highlights the need for implementing more robust somatic calling and variant refinement strategies.

Numerous studies have benchmarked the performance of somatic callers over different sequencing depths and VAFs on paired tumor–normal data [25–29]. There was obvious discordance among tools, especially at low sequencing depth [25,27,28] and on low-VAF variants [25,26]. In fact, a benchmark study by the Pan-Cancer Analysis of Whole Genomes (PCAWG) Consortium revealed that about two-thirds of the discrepancies in mutation calls between WGS and WES from the same cohort are related to low VAFs, and that there is obvious bias from a single caller [30]. Thus, ensemble approaches have been proposed to achieve balance between sensitivity and precision [30–32].

Somatic calling in healthy tissues faces additional challenges. Compared to those in tumor samples, somatic variants in normal tissues typically have much lower VAFs due to lower mutation rates [33]. However, no analytical pipeline has been developed specifically for CHIP detection and refinement. Refinement relying on pre-defined thresholds is less robust and time-consuming. Instead, a few generic callers, mostly developed for mutation detection in cancer genomes, have been used, such as GATK HaplotypeCaller and UnifiedGenotyper [8], VarScan [34], MuTect together with Indelocator [7,10], VarDict [35,36], and Mutect2 [4,37]. Even popular algorithms often lose power on variants with low VAFs [33], including Mutect2 by which majority of the CHIP calls with 2%–5% VAFs were not identified at ∼ 100× sequencing depth [4]. Although reference-free methods have also been developed to minimize reference bias in mutation calling, most of them are optimized for the detection of germline mutation. Two of these methods, Lancet [38] and 2-kupl [39], were developed for the detection of somatic mutations. However, both methods require a matched normal from the same individual, limiting their applicability in CHIP screening.

Also, numerous control-free tools have been developed for the detection of mosaic variants, *i.e.*, genetic mosaicism arising from postzygotic mutations, without requiring a matched control [40–42]. A comprehensive benchmarking study assessed 11 methods derived from 6 callers [43], including MosaicHunter using Bayesian model [40], MosaicForecast based on random forest [42], and DeepMosaic utilizing deep convolutional neural network [41], along with 3 generic germline/somatic callers. The first 3 tools were originally developed for the detection of mosaic variants. The 11 methods were tested with high-coverage data, including 1100× WES-based reference standards [44] and biological data (500× WES and 250× WGS). The comparison revealed that, in single-sample mode, Mutect2 and MosaicForecast showed the best performance at < 10% VAFs. On the other hand, MosaicHunter had high precision but low sensitivity, which could benefit the selection of highly confident variants for validation [43]. However, DeepMosaic had low sensitivity and precision. Importantly, recommended as a top-performing caller by the benchmarking study, MosaicForecast was trained with variants identified by Mutect2 (default) [42], which is known to have low sensitivity on low-VAF CHIP mutations [4]. Among the 3 tools, MosaicHunter and DeepMosaic were not designed for the detection of mosaic insertions and deletions (INDELs) [40,41], and MosaicForecast had poor performance on low-VAF (< 10%) INDELs. This limits their application to the detection INDELs, which account for > 30% of the CHIP mutations [4,7,8]. For WES data, while MosaicHunter has implemented a beta-binomial model to correct capture bias, the other 2 tools are not fully ready for the analysis of WES data, due to a lack of test (MosaicForecast) [42] or a need for further training (DeepMosaic) [41]. As the benchmarking study is based on high-coverage datasets, attention is needed when analyzing low-coverage data. With these limitations, the possible extension of these mosaicism-centered tools to CHIP detection remains to be tested.

To systematically address these issues, this study presents UNIfied SOmatic calling and Machine learning (ML)-based classification, or UNISOM for short, which is a software toolkit designed for streamlined CHIP discovery. UNISOM has implemented a meta-caller to generate an ensemble call set. A ML-based variant classifier was built to separate raw calls into CHIP mutations, germline mutations, and artifacts with little manual intervention. When applied to both simulated and real WES/WGS data, UNISOM demonstrated robust performance over a wide spectrum of sequencing depth and VAF.

## Method

### Leukemia-associated genes and known CHIP mutations

We collected 179 leukemia-associated genes from 4 CHIP studies with WES [7,8,34] or WGS [4]. In addition, we included 189 genes from the Mayo Clinic CHIP diagnostic panel, bringing the total to 202 unique genes (Table S1). A list of 2367 CHIP mutations [1331 single nucleotide variants (SNVs) and 1036 INDELs] was compiled from 3 CHIP studies [4,7,8], as described in “known CHIP mutations” in File S1. Over 90% of the CHIP carriers harbor only a single mutation [4,7,10].

### NA12878 sequencing data

NA12878 is the pilot genome of the Genome in a Bottle (GIAB) consortium, in which a high-confidence germline call set has been generated [45,46]. Such a high-confidence variant set has been widely used in benchmarking variant detection pipelines [47,48]. Also, many WES and WGS data have been generated for NA12878 on multiple sequencing platforms. The use of such diverse data with different sequencing error profiles will minimize bias in tool comparison. This study used 4 internal datasets (2 WES and 2 WGS), as well as BAMs from 5 WES datasets (100×–360× coverage) and 10 WGS datasets (9 at 24×–40× coverage and 1 at 300× coverage) in public sequence repositories [46,49] (Table S2). To reduce computing time and eliminate bias, only alignments falling within the 202 genes were considered. Depending on the original coverage, 6 WES datasets (> 130× coverage) and 3 WGS datasets (> 80× coverage) were downsampled to a coverage series of 200×, 100×, 50×, and 20× (Table S2). Together, 44 BAMs, 19 from WGS and 25 from WES, were used as input for CHIP spike-in.

### CHIP mutation spike-in

We used BAMSurgeon (v1.2) [50] to add the known CHIP variants to each of the aforementioned BAMs, at the same genomic positions where these mutations were originally identified (Figure S1). BAMSurgeon used the default parameter settings, except that “the minimum read depth to make mutation” was set at 2 (m = 2) instead of 10 (default). We simulated 2 batches of BAMs: batch 1 for developing meta-caller and batch 2 for developing ML-based variant classifier (Figure S1). Batch 1 started with 12 BAMs from WES and 9 from WGS (Table S2). Simulation was performed to either have CHIP-specific VAFs, or have the same VAF each time across all the spike-in CHIP variants. With 13 VAF levels (0.5%, 1% to 10% with 1% increment, 20%, and 30%) plus CHIP-specific VAFs, there were 14 simulated datasets varying by VAF, totaling 294 BAMs. Batch 2 was simulated, with CHIP-specific VAFs, from all 44 BAMs (19 WGS and 25 WES). The presence of CHIP spike-in in the output BAMs was verified using bcftools mpileup [51].

### Development of the meta-caller

To maximize CHIP detection, we first developed VarTracker based on the idea of force-calling [12,32,52]. It is a permissive tool that uses bcftools mpileup to track mapped reads along genomic positions for the evidence of alternative base(s). VarTracker has 5 key components: (1) call potential variation sites with bcftools mpileup (“--min-BQ 13”), except in read-dense regions (“-d 1000” by default); (2) use GATK UnifiedGenotyper to genotype candidate variation sites and extract their attributes, such as number of supporting reads, mapping quality, and whether spanning an INDEL site; (3) split multi-nucleotide polymorphisms (MNPs), if any, into individual SNVs; (4) left normalize INDELs; and (5) at multiallelic sites, extract the alternative allele with the most supporting reads, or choose a random one if all alternative alleles have equal number of supporting reads.

To build an ensemble of the most sensitive callers, we benchmarked 10 open-source variant callers (Table S3), together with VarTracker, on the CHIP genomes from batch 1 simulation. The 10 callers include Freebayes [53], GATK Mutect2 [54], GATK HaplotypeCaller [55,56], GATK UnifiedGenotyper [55,56], LoFreq [57], mpileup [51], Platypus [58], Strelka2 [59], VarDict [60], and VarScan2 [61]. These tools were selected as representatives of widely implemented variant detection algorithms [62]. The performance metrics were calculated using the spike-in CHIP as ground truths.


(1) Precision=true positive / (true positive+false positive)

(2) Recall=true positive / (true positive+false negative)

Here, true positive and false negative represent the number of spike-in CHIP variants that were recovered (at their pre-placed positions regardless of the alternative base) and missed by a caller, respectively, while false positive represents the number of variants called from elsewhere that were not part of the spike-in variant set.

To maximize the recall, a meta-caller was developed by combining VarTracker with Mutect2 and VarDict. These tools showed the highest recall, in particular at the low bound of VAFs (0.5%–10%). Of note, Mutect2 and VarDict use haplotype-based strategy (local assembly) and heuristic approach, respectively. VarTracker utilizes bcftools mpileup and GATK UnifiedGenotyper, both of which use a Bayesian genotype likelihood model. Thus, the 3 methods are expected to complement each other in variant detection.

### Development of ML-based variant classifiers

To generate variant training sets for benchmarking ML algorithms in variant classification, meta-caller was used to call raw variants from each of the 44 BAMs in batch 2 that were simulated with CHIP-specific VAFs. Raw variants were annotated with Clinical Annotation of Variants (CAVA) [63], and those predicted to have functional effects were retained.

To build prediction models, the retained variants were assigned with an actual class label of being CHIP, GERMLINE, or ARTIFACT, and split into SNVs and INDELs. Variant-associated features (26 for SNPs and 24 for INDELs) were extracted (Table S4**)**. The mlr R package [64] was used to build 4 ML models, including Recursive Partitioning and Regression Trees (rpart), Support Vector Machine (SVM), random forest, and eXtreme Gradient Boosting (XGBoost), as well as to perform hyperparameter tuning (with *k* = 5) and calculate feature importance. Three data types (WES, WGS, and WES + WGS) were used separately in model building, each with 80% of the raw variants as the training set and the other 20% as the test set. We also tested neural network for CHIP prediction from WGS with TensorFlow platform (https://www.tensorflow.org) in Python environment. Key steps are illustrated in Figure S1 and described with more details in “ML-based variant classifier” in File S1.

In calculating the performance metrics, the pre-assigned actual class labels were used to represent the actual class labels. For a given model, its predictive performance was assessed by evaluating the 3-class assignments (*i.e.*, CHIP, GERMLINE, and ARTIFACT) of variants made on the test set against the actual class labels. The prediction outcome was summarized as a 3 × 3 confusion matrix, which shows the number of raw variants, from a given actual class, that fall into each of the predicted classes. Next, for CHIP, the prediction sensitivity (recall, Equation 2), specificity (Equation 3), precision (Equation 1), F1 score (*i.e.*, harmonic mean of precision and recall, Equation 4), and accuracy (Equation 5) were calculated based on the confusion matrix.


(3) Specificity=true negative / (true negative+false positive)

(4) F1 score=2×(Precision×Recall) / (Precision+Recall)

(5) Accuracy=(true positive+true negative) / (true positive+true negative+false positive+false negative)

Here, true positive and false negative are the numbers of actual CHIP variants that are predicted as CHIP and non-CHIP (*i.e.*, GERMLINE and ARTIFACT), respectively. True negative and false positive represent the numbers of non-CHIP variants that are predicted as non-CHIP and CHIP, respectively. For GERMLINE prediction, the metrics were calculated similarly.

### Prediction refinement

XGBoost and random forest were selected based on the performance metrics. Given the typical coverage of WES/WGS, there is a risk that a small fraction of true CHIP variants might have been erroneously predicted as non-CHIP variants by XGBoost and random forest. Therefore, we implemented 2 rules to rescue these predictions. First, non-CHIP variants are flagged as putative CHIP variants if reported in previous studies [4,7,8] and also detected here by at least two of the callers in our workflow. Second, CHIP variants typically have ≤ 30% VAF [8,65], and variants with higher VAFs are classified as germline by default in our workflow. However, a putative CHIP prediction will be assigned for further review if identified in the 5 genes (*DNMT3A*, *ASXL1*, *TET2*, *PPM1D*, and *JAK2*), as germline variants have rarely been identified in these genes [8].

Following the prediction refinement, a hierarchical Bayesian model was developed to estimate confidence intervals (CIs) for CHIP predictions (File S2). With WES from 75 healthy individuals younger than 40 years of age, the model utilizes CHIP incidence in this reference population as the background (see “Confidence interval estimation” in File S1).

### XGBoost prediction in WES

The performance of meta-caller and XGBoost classifier was further evaluated on real WES data from a cohort of 25 individuals, for whom targeted deep sequencing data (∼ 1000× median coverage) were also available. WES had a median coverage of 158× (127×–179×). Variants were detected with meta-caller, at default parameter setting, followed by functional annotation with CAVA. Those predicted to have functional effects were used as input for the XGBoost classifier pre-trained on simulated WES data. To assess the prediction, we used the 33 CHIP variants identified from targeted sequencing data as ground truths (see “CHIP prediction with WES data” in File S1). We used recall, based on Equation 2, to measure the predictive performance, where true positive and false negative are those from the 33 mutations that are detected and missed by UNISOM, respectively.

**Figure 1:**
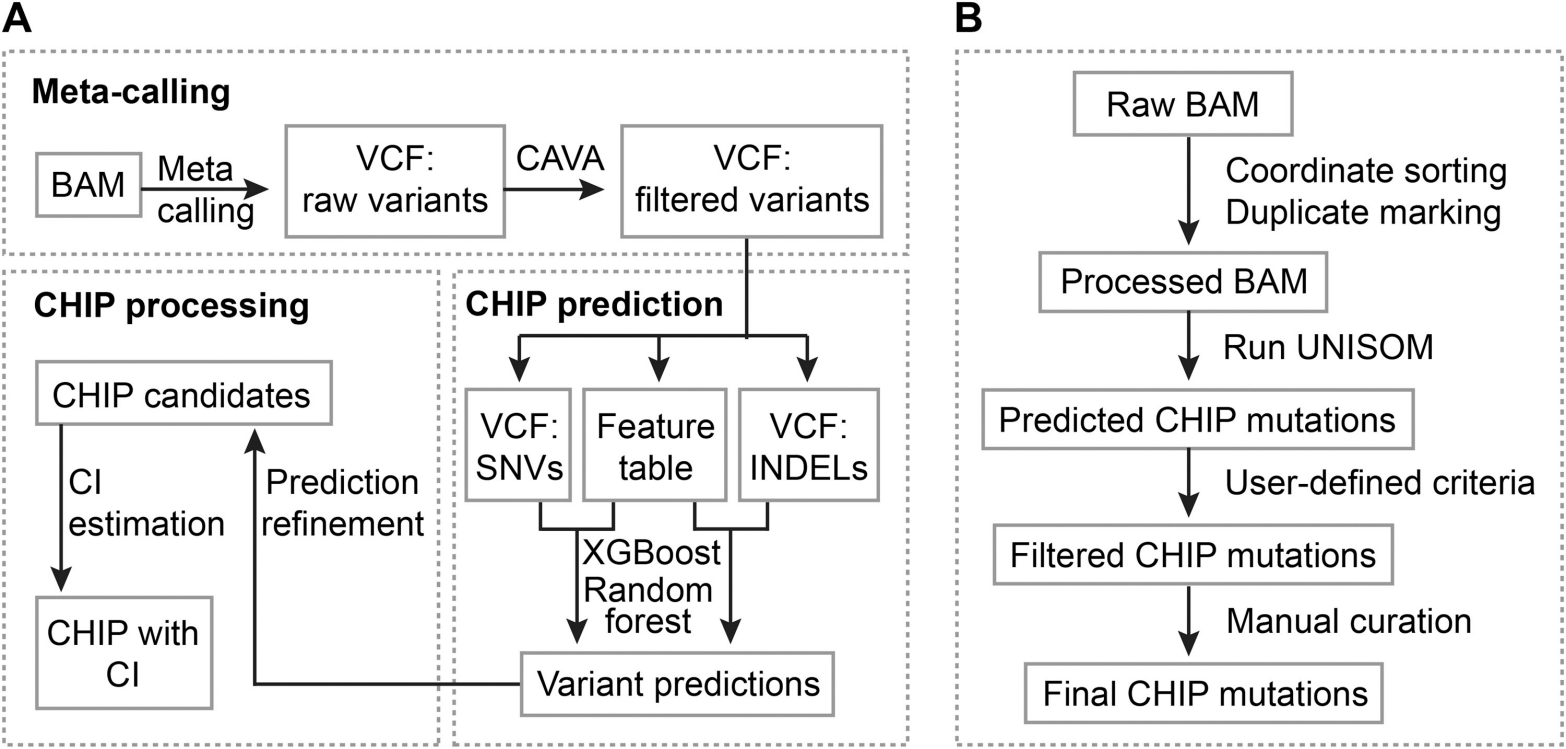
CHIP prediction with UNISOM pipeline **A**. Overview of UNISOM pipeline. The pipeline has 3 major components: meta-calling, ML-based prediction, and CHIP processing. Meta-caller is an ensemble of Mutect2, VarDict, and VarTracker. It merges variants from the 3 tools and assigns a status of 1 to 3 to indicate the number of tools identifying a given variant. Raw variants predicted to have functional effects by CAVA, along with the associated features, are used as the inputs for XGBoost or random forest prediction. These features, 26 for SNVs and 24 for INDELs, cover variant quality metrics, calling status, and genomic context, as well as overlap with public variant databases. The prediction model was trained separately for WES *versus* WGS and for SNV *versus* INDEL. The predictions based on XGBoost or random forest model are subjected to further refinement and prioritization. The refinement step recovers those that are most likely to be erroneously predicted as non-CHIP for manual inspection. To enable prioritization of the predicted CHIP, a hierarchical Bayesian model is used to estimate their CIs. The model utilizes CHIP incidence in a population of healthy individuals (here with age < 40 years) as the background. **B**. Key steps recommended for CHIP discovery. To run UNISOM, raw BAM is pre-processed with Picard, which includes coordinate sorting, duplicate marking, and filtering based on mapping quality. UNISOM predicted CHIP mutations are then filtered, such as removal of common variants and extraction of subset based on user provided lists of leukemia driver genes and driver mutations. Finally, to rule out the possibility of being alignment artifacts, it is often necessary to check alignments around the retained variants in a genome browser. CAVA, clinical annotation of variants; CHIP, clonal hematopoiesis of indeterminate potential; CI, confidence interval; XGBoost, eXtreme Gradient Boosting; INDEL, insertion and deletion; ML, machine learning; SNV, single nucleotide variant; VCF, variant call format; WES, whole-exome sequencing; WGS, whole-genome sequencing.

Based on the aforementioned analysis of simulated and WES data, we developed the UNISOM pipeline, which includes meta-calling, XGBoost or random forest prediction, CHIP prediction refinement, and CI estimation (**Figure 1**A).

### CHIP prevalence in Mayo Clinic Biobank

To demonstrate its utilization, we applied UNISOM to the ∼ 30× WGS data from 979 individuals in the Mayo Biobank [66]. DNA derived from whole blood was used in WGS. Library preparation and data processing were reported [67]. Prior to CHIP screening, data which failed to meet the sequencing quality metrics and the relatedness check or from participants who self-reported a hematologic malignancy diagnosis in the questionnaire were excluded. UNISOM used default parameters, with the XGBoost classifier trained on WGS data.

Predicted CHIP mutations were removed if: (1) with ≥ 0.3% minor allele frequency (MAF) in any of the 4 germline databases (Table S4), or with > 30% VAF unless identified within the 5 leukemia driver genes (*DNMT3A*, *ASXL1*, *TET2*, *PPM1D*, and *JAK2*) [8] (see “Prediction refinement” section above); (2) low-confidence variants within low-complexity genomic regions [68]; or (3) found in ≥ 8% of the subjects that likely represent technical artifacts as proposed in [69]. The other variants with ≥ 2% VAF and present in the Catalogue Of Somatic Mutations In Cancer (COSMIC) database were retained. To enable comparison with previous cohort studies, we limited our analysis to a list of leukemia driver mutations [4,7], available in Supplementary Table 2 from [4].

## Results

### Overview of UNISOM pipeline

To predict CHIP variants, UNISOM integrates variant calling and annotation, as well as CHIP prediction, refinement, and CI estimation in a seamless framework (Figure 1A). It uses BAMs as input and predicts CHIP in a list of leukemia-associated genes. The pipeline includes 5 steps**:** (1) variant calling with a meta-caller for high sensitivity; (2) variant annotation for quality, genomic context, prevalence in the general population, and impact on protein function using CAVA; (3) classification of annotated variants into CHIP, GERMLINE, and ARTIFACT using XGBoost- or random forest-based model; (4) rule-based refinement to rescue potential CHIP variants from possible mis-predictions (as non-CHIP); and (5) optionally, CI estimation for the predicted CHIP using a hierarchical Bayesian model. We outlined the procedure when applying UNISOM pipeline, from raw alignments to CHIP mutations (Figure 1B).

### Benchmarking of single-sample variant callers on simulated data

To benchmark single-sample variant callers in CHIP detection, particularly at low VAFs (0.5%–10%), we simulated a total of 294 CHIP genomes in NA12878 using 13 uniform VAFs, as well as CHIP-specific VAFs by replicating known CHIP mutations. These known mutations have a median VAF of 12.5% from 1331 SNVs and 14.8% from 1036 INDELs. By counting the pileup at genomic positions pre-inserted with these known CHIP variants, we checked whether data simulated with CHIP-specific VAFs can capture their actual VAF spectrum. Using sample NA12878_01 as an example (Table S2), the SNVs and INDELs had a median VAF of 5.4% and 4.7%, respectively, in the simulated 50× WES data, similar to that in simulated 50× WGS data (4.8% and 6.3%). The trend of lower VAFs, compared to those of the actual spike-in CHIP variants (Figure S2), was observed in all simulated data. This discrepancy is partly due to the fact that not all CHIP mutations have been successfully simulated into the genome, given the low VAFs of many CHIP mutations and insufficient depth of coverage. Of note, the overall lower VAFs in simulated data do not prohibit the benchmarking of callers that have higher sensitivity at low VAFs.

To identify the most sensitive callers, we used the alignments (BAMs) simulated with uniform VAF. Overall, the tools performed comparably in the detection of germline variants; however, marked differences were observed in the detection of somatic mutations. Using the highly confident germline calls (NISTv3.3.2) [46] as the ground truths, all the 11 tools had ≥ 96% SNV recall rate in 100× WGS and 10 tools (except VarScan, 92% recall rate) had ≥ 98% recall rate in 100× WES from NA12878_01. For the detection of spike-in CHIP variants, VarTracker had the highest recall for both SNVs and INDELs, followed by VarDict and Mutect2, over the full range of VAFs tested (Figure S3A–D**)**. VarTracker identified 65.7%–85.4% of the spike-in SNVs, *versus* only 27%–54.5% by VarDict and Mutect2 (Figure S3A and B). A similar pattern was observed for INDELs (Figure S3C and D). However, the remaining 8 callers tested had much reduced recall, more obviously at < 10% VAFs.

**Figure 2:**
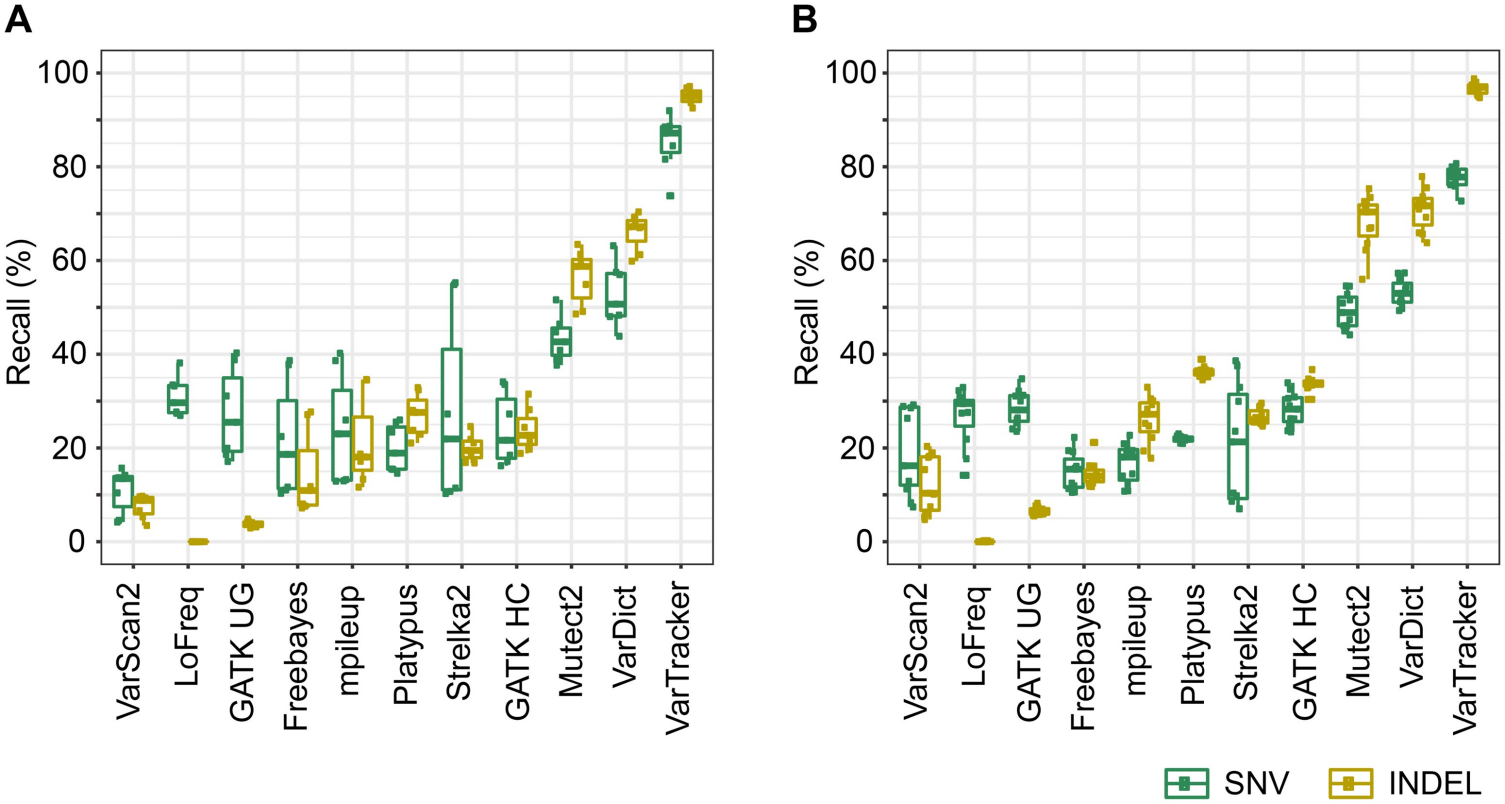
Recall of 11 tools tested on simulation data **A**. Simulated SNVs and INDELs in WES. **B**. Simulated SNVs and INDELs in WGS. Each box plot in (A) and (B) uses recall rates estimated from 7 WES and 11 WGS data, respectively, split into SNV and INDEL. All data are from batch 1 simulation that uses CHIP-specific VAFs, excluding those with > 100× coverage. The 7 WES data are from datasets 13, 18, and 19 with coverage of 20×, 50×, and 100×, while the 11 WGS data are from datasets 1, 3, 4, 11, and 12 with coverage of 20×, 29×, 50×, 84×, and 100× (Table S2). VarTracker, VarDict, and GATK Mutect2 showed the highest recall for both SNVs and INDELs. GATK HC, GATK HaplotypeCaller; GATK UG, GATK UnifiedGenotyper; VAF, variant allele frequency.

Next, we evaluated variant calling performance with simulated data at different read depths (20×–100×) **(****Figure 2**A and B, Figure S4A and B). VarTracker, VarDict, and Mutect2 had the highest recall for both SNVs and INDELs in WES (Figure 2A) and WGS (Figure 2B) across all depths. Combining WES and WGS for simplicity, VarTracker had a recall of 72.7%–92% for SNVs and 92.5%–98.8% for INDELs, which was 21%–44% higher than that of VarDict and Mutect2. Not surprisingly, of the 3 callers, Mutect2, which had the lowest recall, had the highest precision, followed by VarDict and VarTracker (Figure S4A and B). Previous studies have reported a dependence of calling sensitivity on coverage depth for VarDict [70] and Mutect2 [4], as well as SAMtools mpileup (*i.e.*, bcftools mpileup) and GATK UnifiedGenotyper (both used by VarTracker) [71]. Thus, we also checked how coverage impacts variant calling for these methods. As expected, overall, the number of identified spike-in variants increased with coverage for both WES (Figure S5A and B) and WGS (Figure S5C and D).

**Figure 3:**
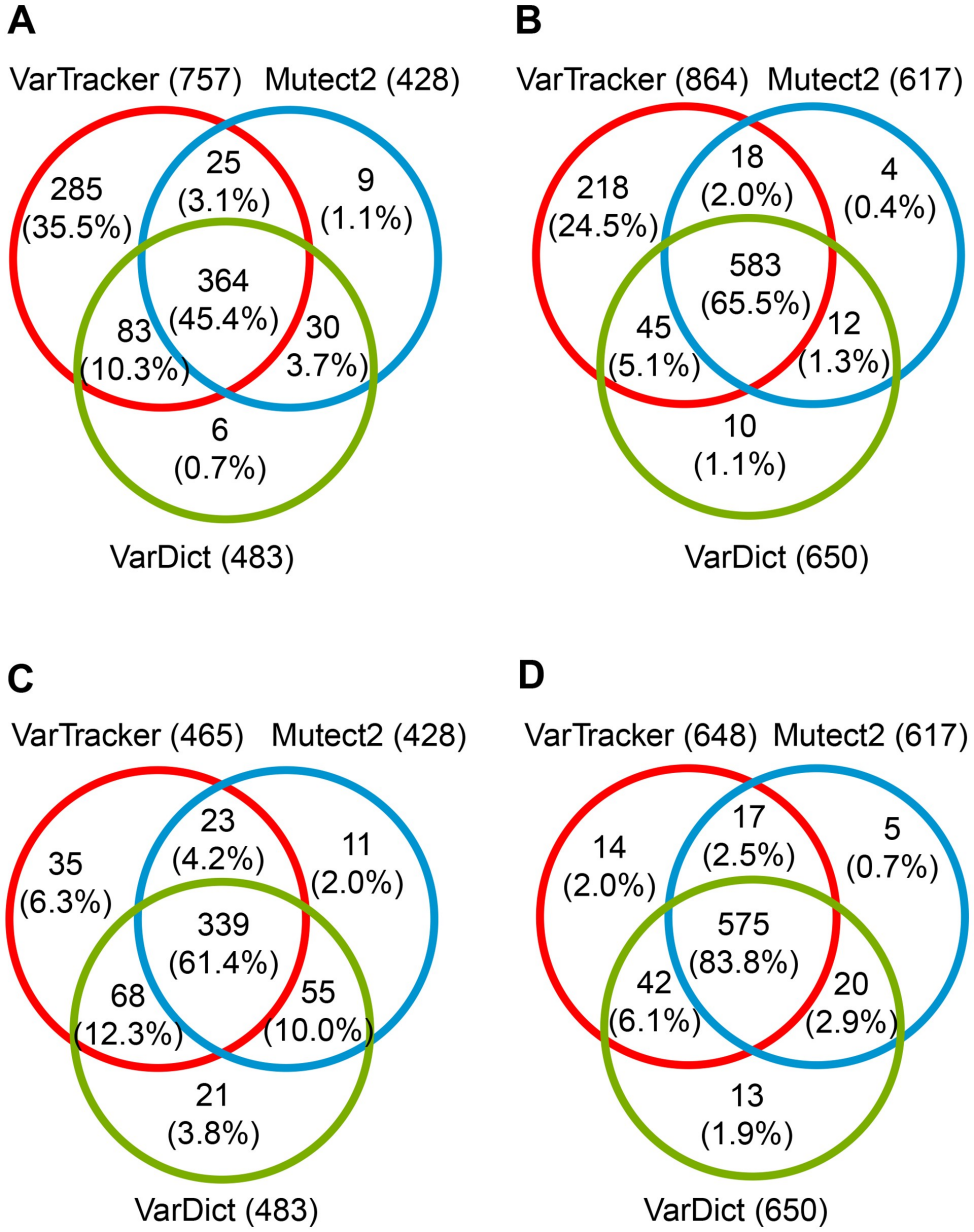
Overlap of spike-in CHIP variants recovered by the three callers **A**. NA12878_02 WES simulated at 100× coverage. At least 1 read supports alternative allele. **B**. NA12878_02 WGS simulated at 50× coverage. At least 1 read supports alternative allele. **C**. The same WES data as in (A). At least 2 reads support alternative allele. **D**. The same WGS data as in (B). At least 2 reads support alternative allele. Both data are simulated with CHIP-specific VAFs. In each plot, SNVs and INDELs are combined. While VarTracker can report variants with a single supporting read, Mutect2 and VarDict only output those with ≥ 2 supporting reads.

The aforementioned analyses revealed that VarTracker, VarDict, and Mutect2 had the highest recall in the simulated data. Of note, the increased recall from VarTracker is mainly due to the inclusion of calls with a single supporting read, which were not reported by Mutect2 and VarDict. To support this, we checked overlap of the 3 tools in recovering spike-in from 100× WES (**Figure 3**A and C) and 50× WGS data (Figure 3B and D) in sample NA12878_02 (Table S2). Of the total spike-in, about 90% were recovered by the 3 tools combined (Figure 3A and B), which dropped to < 70% if excluding VarTracker calls with a single supporting read (Figure 3C and D). In fact, about 90% of the VarTracker unique calls (not identified by the other two) had only a single supporting read (Figure 3A and B). At the same cutoff of ≥ 2 supporting reads, VarTracker and VarDict had comparable recall, which was 3%–7% higher than that of Mutect2 (Figure 3C and D). To enhance the variant detection, a meta-caller was built by combining the 3 tools.

### ML-based variant classification in simulated data

It remains challenging to distinguish CHIP mutations from germline variants solely based on VAFs, especially for those occurring in large, mutated clones. Furthermore, the 3 tools selected for meta-calling, VarTracker in particular, had high recall but low precision based on the simulated data (Figure S4A and B). We hypothesized that, by learning the underlying features of known CHIP mutations *versus* germline variants and artifacts, ML approaches could filter out most of the non-CHIP variants, thus minimizing the manual inspection needed in variant refinement. Toward this, we first assessed the predictive performance of 24 ML models on batch 2 data simulated with CHIP-specific VAFs (Table S2), involving 4 ML algorithms, 2 variant types (SNVs and INDELs), and 3 data types (WGS, WES, and combined data) (Figure S1).

At default parameter setting, compared to the other two, XGBoost and random forest showed higher accuracy, recall, precision, and F1 scores across nearly all models in CHIP prediction (**Table 1**). For XGBoost and random forest (and the other two as well), the predictive performance is clearly better on INDELs than on SNVs, showing 0.1–0.2 increase in F1 score, 17%–33% increase in recall, and 6.2%–14.4% increase in accuracy (Table 1). Not surprisingly, both methods showed better performance in WGS compared to WES, which is likely due to the greater bias observed in the latter (*e.g.*, less uniform coverage stemmed from variation in capture efficiency and greater GC bias) [72]. Consequently, it is known that WGS is more powerful in identifying exonic variants than WES [73]. Nevertheless, hyperparameter tuning showed negligible gains over the default parameters in overall performance (Figure S6A–C).

**Table 1:** Performance metrics of ML algorithms in predicting CHIP

Data	Algorithm	SNV				INDEL				
		F1 score	Recall (%)	Precision (%)	Accuracy (%)		F1 score	Recall (%)	Precision (%)	Accuracy (%)
WES	SVM	0.642	50.6	87.7	75.2		0.898	86.2	93.8	90.1
	rpart	0.650	50.2	92.2	75.1		0.927	50.2	92.9	92.7
	Random forest	0.763	63.0	96.7	81.5		0.960	96.4	95.7	95.9
	XGBoost	0.766	64.8	93.8	82.4		0.961	96.5	95.7	95.9
WGS	SVM	0.752	62.2	95.2	81.0		0.937	94.5	92.9	91.3
	rpart	0.732	59.7	94.6	79.8		0.944	95.5	93.3	91.3
	Random forest	0.863	78.5	95.9	89.2		0.970	97.6	96.4	95.7
	XGBoost	0.875	80.7	95.6	90.2		0.975	97.8	97.3	96.4
WES + WGS	SVM	0.691	55.4	91.8	77.6		0.914	90.4	92.4	90.3
	rpart	0.681	52.4	97.1	76.2		0.929	94.7	91.1	91.2
	Random forest	0.817	71.0	96.3	85.5		0.967	96.9	96.5	96.0
	XGBoost	0.832	74.2	94.7	87.0		0.966	96.9	96.4	96.2

*Note*: Accuracy, F1 score, recall, and precision are estimated from confusion matrix, using Equations 5, 4, 2, and 1, respectively. Overall, XGBoost and random forest had higher accuracy, F1 score, recall, and precision compared to SVM and rpart. The four ML algorithms were each assessed on the 2 variant types (SNVs and INDELs) and 3 data types (WGS, WES, and combined). Data are from batch 2 simulation with CHIP-specific VAFs (Table S2). CHIP, clonal hematopoiesis of indeterminate potential; XGBoost, eXtreme Gradient Boosting; INDEL, insertion and deletion; ML, machine learning; rpart, Recursive Partitioning and Regression Trees; SNV, single nucleotide variant; SVM, Support Vector Machine; WES, whole-exome sequencing; WGS, whole-genome sequencing.

Similarly, XGBoost and random forest also had higher accuracy, recall, and precision in predicting germline SNVs, while in predicting germline INDELs, SVM also had comparable performance (Figure S7A–F). Thus, XGBoost and random forest models were selected as the variant classifiers, with the former having slightly higher F1 score (due to the increased recall) in SNV detection from WGS.

We also assessed the performance of neural network model on WGS data from batch 2 simulation with CHIP-specific VAFs. Compared to XGBoost and random forest, the neural network model showed 7.3%–16.1% decrease in recall, as well as 5.3%–9.1% decrease in accuracy and precision (Table S5). Thus, neural network was not implemented for CHIP prediction (Figure 1A).

**Figure 4:**
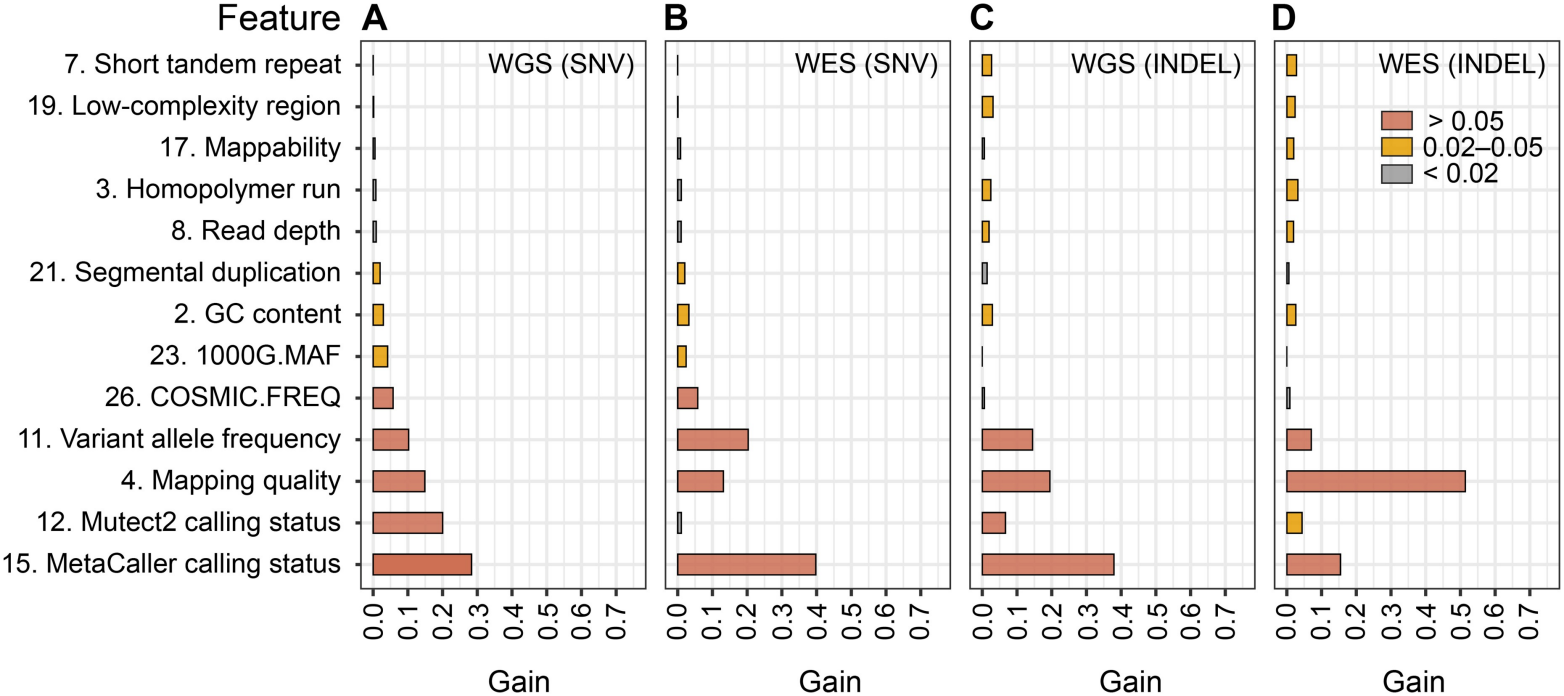
Contributions of the top 13 features to the predictive performance of XGBoost **A**. Feature’s gain in predicting SNVs from WGS. **B**. Feature’s gain in predicting SNVs from WES. **C**. Feature’s gain in predicting INDELs from WGS. **D**. Feature’s gain in predicting INDELs from WES. The gain value is calculated using the xgb.importance function within the mIr package, which indicates a feature’s relative contribution to the model. The 13 features are selected to have gain values of ≥ 0.02 in at least 1 of the 4 predictions, showing in ascending order based on (A). The number before each feature is from the “Feature” column in Table S4. 1000G, 1000 Genomes Project; MAF, minor allele frequency; COSMIC, Catalogue Of Somatic Mutations In Cancer; FREQ, number of samples carrying the variant in the COSMIC database.

In XGBoost model, WGS was moderately correlated with WES in SNV [Pearson correlation (*r*) = 0.82] and INDEL (*r* = 0.64**)** classification based on all features (Table S4). To understand which of the features contribute most to the overall CHIP prediction and how their contributions vary by sequencing platform and variant type, we analyzed feature importance in XGBoost classifier. For simplicity, we plotted the top 13 features, each having ≥ 0.02 gain in at least one of the variant sets (**Figure 4**A–D). Overall, meta-calling status (*i.e.*, number of callers identifying a variant), mapping quality score, and VAF represent the most important features across all the 4 variant sets, together achieving a gain of 0.54–0.74.

Individually, the magnitude of contribution from each feature appears to be sequencing- and variant type-dependent. For example, in predicting INDELs (Figure 4D), mapping quality alone had > 0.5 gain in WES, *versus* < 0.2 in the other 3 variant sets. This observation recapitulates the essence of accurate alignments in INDEL discovery, especially for WES that is known to enrich with low-quality INDEL calls compared to WGS [74]. In addition, Mutect2 calling status had larger contribution to variant classification in WGS compared to WES, supporting that the local assembly strategy implemented in Mutect2 has greater power in WGS due to its more uniform and continuous read coverage. Third, COSMIC match had noticeable contributions to the detection of SNVs (gain > 0.05), but much less for INDELs (gain < 0.01), likely owing to the overrepresentation (∼ 7-fold) of SNVs in the database. On the other hand, homopolymer run, low-complexity region, and short tandem repeat impacted the classification of INDELs, but less so for that of SNVs (Figure 4C and D). Indeed, these 3 elements are known to be associated with high false positive calls [68,74] or high mutation rates of INDELs [75]. Taken together, our analyses demonstrate that XGBoost model can reliably predict CHIP over a wide range of VAFs, and that the prediction power is primarily attributed to a few associated features of high importance.

### Pipeline validation with WES data

We applied UNISOM to real WES data from a cohort of 25 subjects with a confirmed absence of cytopenias or other hematologic disorders. To assess its performance, CHIP mutations were also identified from the same cohort with targeted deep sequencing (Table S1). Specifically, from targeted panel, a total of 45 CHIP variants (only including SNVs due to very few INDELs) were retained after manual checking. In WES, 5 of these were mapped outside of the capture regions and another 7 had no coverage, leaving the other 33 (including 12 known CHIP variants) as ground truths.

WES data had an average of 158× coverage, with approximately 90% of the regions having at least 20× coverage. For each subject, meta-caller identified 18,080 raw variants on average, from which 11 were predicted as CHIP based on ML models trained on WES and WES + WGS, and 13 as CHIP based on WGS model. The vast majority (> 99.9%) of the raw variants were classified as artifacts. The result indicates that, by automatically eliminating the artifacts, our approach could substantially reduce the manual work to refine CHIP candidates.

For the 33 CHIP mutations (“ground truths”) identified by targeted sequencing, the 3 models predicted 26–27 of them as CHIP in WES, including all the 12 known CHIP mutations previously reported [4,7,8], with 76.5%–79.4% recall (**Table 2**). These CHIP mutations each had at least 2 supporting reads, with 5.6%–51.4% VAF for 22 and 1.3%–4.1% VAF for the other 5 mutations. The remaining 6 CHIP mutations were mis-predicted as artifacts; 4 of the 6 were identified by VarTracker alone with a single read supporting, failing to meet the cutoff of ≥ 2 supporting reads, while the other 2 were identified by VarTracker and VarDict but had low (< 3%) VAFs. We also tested how sequencing depth impacts the detection of CHIP mutations by using two-thirds and one-third of the alignments after downsampling. Applying ML-based prediction model pre-trained on WES data, we recovered all 27 CHIP mutations with two-thirds of the alignments. However, with one-third of the alignments, only 22 CHIP mutations were recovered. Of the 5 CHIP mutations missed, all had only 2–4 reads supporting the alternative allele and 4 of the 5 had low VAFs of 3.5%–5.7% at two-thirds of the alignments. Thus, mutations with low VAFs and few supporting reads are more sensitive to the reduction of sequencing depth.

**Table 2:** Prediction of CHIP in WES data

ML model	CHIP	Germline	Artifact	Recall (%)
WES	27	0	6	79.4
WES + WGS	27	0	6	79.4
WGS	26	1	6	76.5

*Note*: The 33 CHIP mutations identified by deep targeted sequencing from the same cohort were used as the ground truths. The 3 ML-based models correctly predicted 26–27 of them as CHIP in WES. The WGS model erroneously predicted 1 CHIP mutation (on chrX with 42.7% VAF) as germline. Twelve of the 26–27 predicted CHIP mutations overlapped those discovered previously. VAF, variant allele frequency.

### CHIP discovery in Mayo Clinic Biobank

To demonstrate UNISOM application in large-scale cohort studies, we analyzed WGS data (∼ 30×) from 979 participants in the Mayo Clinic Biobank who self-reported no diagnosis of hematological malignancies. Briefly, variants were identified using meta-caller at default parameter setting, followed by CHIP prediction on CAVA-annotated variants using the WGS-trained classifier. In comparison with previous CHIP studies using WES [7] and WGS [4], our analysis also focused on the leukemogenic driver mutations used in both studies [4,7].

On average, Mutect2, VarDict, and VarTracker identified 110 (85–136), 171 (93–265), and 1888 (420–10,777) raw variants per subject. Based on ML-based prediction models trained on WGS, the vast majority of the raw variants were classified as artifacts or germline variants and were filtered out. Only 1–3 CHIP mutations were predicted per subject in 17.5% (171) of the subjects. A total of 202 CHIP mutations were identified in 35 driver genes at ≥ 2% VAF (Table S6), with at least 2 reads supporting the alternative allele. All the 35 genes are included in the Mayo CHIP gene panel and TOPMed study [4]. Two-thirds (133/202) represented mutations discovered in previous CHIP studies [4,7,8].

**Figure 5:**
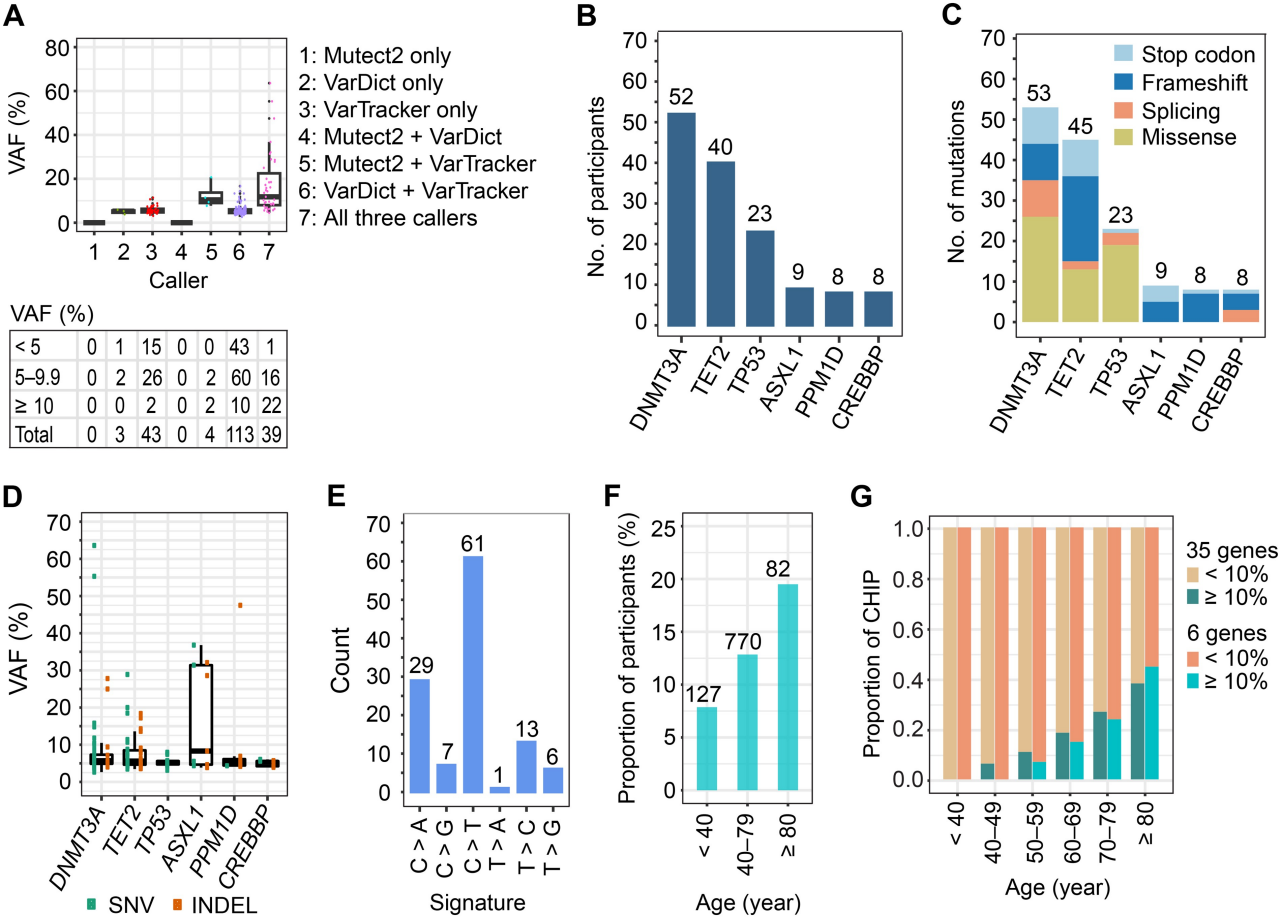
CHIP mutations in Mayo Biobank cohort **A**. VAFs of CHIP mutations. The bottom and upper lines in the box plot represent the 25% and 75% percentiles, respectively, with the horizontal line within representing the median. CHIP mutations are separated based on the tool(s) identifying them. The table at the bottom lists the number of CHIP mutations only identified by each of the 3 tools and by their 4 combinations, separated by VAF. No CHIP mutation is detected by Mutect2 alone (“Mutect2 only”) or by Mutect2 plus VarDict (“Mutect2 + VarDict”). Of the 3 tools, VarTracker is most sensitive at low VAFs of < 10%, with 41 (15 + 26) unique calls. VarTracker and VarDict both identify CHIP mutations at VAF down to 2.6%, while Mutect2 reaches 4.5% VAF. **B**. CHIP prevalence in the top 6 genes with the most mutations. For each gene, Y-axis shows the number of participants who carry at least 1 CHIP mutation in that gene. **C**. Number of CHIP mutations in the 6 genes. The total mutations per gene are split by mutation type. For each gene, if two or more mutations occur in the same individual, or if the same mutation occurs in two or more individuals, they are summed up. **D**. VAFs of CHIP mutations in the top 6 genes. No INDEL is identified in *TP53*. **E**. Signatures enriched in CHIP mutations. **F**. Prevalence of CHIP mutations in 3 age groups. There is a trend of increased CHIP prevalence by age as previously reported. **G**. VAF *versus* age of CHIP carriers. Considering all the 35 mutated genes and the top 6 genes, there is a clear trend that the proportion of “large” mutated clones (VAF ≥ 10%) increases with age.

We first examined the VAF distribution stratified by caller (**Figure 5**A). Both known and novel mutations had similar median VAF (5.6% *versus* 5.9%), which is about 3-fold lower than that from WGS-based TOPMed study [4]. Looking closely at the clonal fraction, 82% of the 202 mutations had low VAFs of < 10%. Among these low-VAF mutations, nearly 90% were identified by VarTracker alone or together with VarDict, but missed by Mutect2 (Figure 5A). The result supports the estimation that, for 30× WGS (equivalent to the average coverage in Mayo Biobank cohort), Mutect2 would miss about 90% of the CHIP mutations with < 10% VAFs [4]. Our analysis demonstrates the high sensitivity of the meta-calling strategy toward low-VAF mutations, even from data without high sequencing coverage.

We next examined the CHIP prevalence in this cohort. The results showed that 17.5% (171/979) of the participants carried CHIP mutations (Table S6), which is much higher than the proportions reported in previous WES [7] and WGS studies [4]. The high CHIP prevalence observed here is largely attributed to the meta-calling. Indeed, if only considering mutations identified by Mutect2 (Figure 5A), CHIP prevalence dropped to 3.9%, at a similar level (4.3%) as the TOPMed WGS project (∼ 40×) that used Mutect2 [4]. Of the CHIP carriers, 143 individuals each had a single mutation. The other 28 subjects each had 2–3 mutations in 16 genes (Figure S8**)**, 10 of which previously were reported with co-mutations [7]. Among the 979 subjects, 5 did not provide the hematological phenotype status in the questionnaire. CHIP mutation was identified in only one of them, at 6% VAF within *EZH2*. Thus, the high CHIP prevalence observed in this cohort is unlikely due to the presence of cancer cells in blood.

CHIP mutations are known to be highly enriched in a few driver genes, including *DNMT3A*, *TET2*, and *ASXL1* that together harbored ∼ 70%–80% of the mutations [4,7,8]. Similar enrichment was observed in the Mayo Biobank cohort, with the top 6 genes carrying > 70% of the mutations (Figure 5B and C). However, for the 6 genes, Mutect2 missed 28.6% of the known CHIP mutations in *ASXL1* and 75.5%–100% in the other 5 genes. Five of them (except *CREBBP*) are among the 8 most frequently mutated genes [4,8].

In previous studies [4,7], *CREBBP* is not one of the most commonly mutated CHIP genes. In the TOPMed cohort, Mutect2 identified only 7 CHIP mutations in *CREBBP*, compared to 357 in *ASXL1* [4]. All mutations in *CREBBP* and 93% in *ASXL1* had ≥ 10% VAFs. In Mayo Biobank, however, we identified a comparable number of CHIP mutations in *ASXL1* (7 known and 2 novel) *versus CREBBP* (7 known and 1 novel), attributed to the high sensitivity of our pipeline. In *ASXL1*, 5 of the mutations (8.3%–36.8% VAF) were identified by all the 3 callers, and the other 4 with lower VAFs (≤ 6%) were only identified by VarDict and/or VarTracker. The c.1934dupG mutation was identified in 2 subjects with > 28% VAF. Initially considered as being PCR artifacts, this variant was later confirmed to represent bona fide somatic mutation in hematologic malignancies based on Sanger sequencing and superRCA assay [76,77]. Also, this variant was believed to be true CHIP mutation, supported by the observation in UK Biobank that large-clone (VAF ≥ 10%) c.1934dupG mutation was associated with nearly 20-fold increase in the risk of incident myeloid cancer [24]. In contrast, all the mutations (VAF ≤ 6%) in *CREBBP* were missed by Mutect2 but identified by both VarDict and VarTracker. For the same reason, in *TP53*, there is a higher prevalence of CHIP mutations in Mayo Biobank compared to the previous studies [4,7,8], where none of the mutations in Mayo Biobank were identified by Mutect2. The results support the previous observation that Mutect2 had low sensitivity toward low-VAF mutations [4]. Further, there was heterogeneity of clonal fraction within 4 of the 6 genes (*DNMT3A*, *TET2*, *ASXL1*, and *PPM1D*) (Figure 5D). For example, the median clonal fraction in *ASXL1* (∼ 8.3%) is higher than that of the other 5 genes (5.2%–5.7%), a trend previously reported [4].

We examined the type of mutations in the 6 genes (Figure 5C), which revealed similar portions of disruptive *versus* missense mutations, as previously reported [7,8]. In *TP53*, majority (∼ 60%–80%) of the mutations are missense mutations [7,78,79], a pattern we also revealed in this cohort. In contrast, these 4 genes (T*ET2*, *ASXL1*, *PPM1D*, and *CREBBP*) predominantly or exclusively carried disruptive mutations, such as nonsense, frameshift, and essential splice-site mutations (Figure 5C), consistent with previous findings [7,8]. Finally, within *DNMT3A*, about half (51%) of the mutations are disruptive mutations, a proportion comparable to that (49%) from [7]. As previously found in CHIP mutations [4,7], the most common single nucleotide substitution is cytosine to thymine (C > T) transition (52%), a signature known to be associated with aging [80,81], followed by cytosine to adenine (C > A) transversion and thymine to cytosine (T > C) transition (Figure 5E).

We analyzed factors known to be associated with CHIP prevalence in the earliest CHIP studies, including age [7,8] and smoking status [8]. In this cohort, considering the 6 most commonly mutated genes, CHIP prevalence was strongly correlated with age at the time of sample collection (*P* = 7.7E–03), from 8% for subjects < 40 years of age to nearly 20% among subjects ≥ 80 years of age (Figure 5F). From subjects of < 50 years of age, UNISOM identified 31 CHIP mutations (Table S6). However, Mutect2 identified none of them. Mutect2 also missed 90% of the CHIP mutations in subjects of 50–59 years of age. These results suggest that using Mutect2 along will lead to biased view about the prevalence of CHIP with age. Further, previous studies of longitudinal blood samples predicted that about half of the mutated clones continue to grow over time [9,17], which indicates that the old individuals should have more large-clone CHIP mutations compared to the young individuals. To confirm this in our cohort, using VAF as a surrogate for clone size, we counted small-clone (VAF < 10%) and large-clone (VAF ≥ 10%) mutations, based on the VAF criterion used in [13,36], and plotted their proportions over 6 age groups (Figure 5G). Indeed, for CHIP mutations in the 6 genes, there is a strong accumulation of large-clone mutations over age, from 0% for individuals of < 50 years of age to 44% among those of ≥ 80 years of age. Analysis of CHIP mutations in all the 35 mutated genes revealed a similar trend.

The association with smoking was first identified in a cohort of ∼ 13,000 subjects [8]. With a sample size of < 1000 subjects in Mayo Biobank cohort, smoking was also found to be associated with increased CHIP prevalence [odds ratio (OR) = 1.47, 95% CI: 1.05–2.07, *P* = 0.026] after adjusting for age in the multivariate logistic regression model. To understand how the 3 mutation callers in UNISOM may impact the discovery of association, we repeated the analysis using 3 subsets of CHIP mutations. Subset 1 includes those identified by Mutect2. Subset 2 includes the remaining mutations that were not identified by Mutect2 (73% of them by VarDict and 98% by VarTracker). Subset 3 includes mutations rescued in the refinement step, which are known CHIP mutations initially predicted as “non-CHIP” variants. They were identified by both VarDict and VarTracker, but not by Mutect2. The association with smoking remained significant for Mutect2-missed mutations in subset 2 (OR = 1.55, 95% CI: 1.06–1.55, *P* = 0.024), of which all but 3 were identified by VarTracker, and for the rescued mutations in subset 3 (OR = 2.23, 95% CI: 1.28–2.23, *P* = 0.0047). However, with only CHIP mutations identified by Mutect2 (subset 1), the association with smoking was no longer significant (OR = 1.22, 95% CI: 0.62–1.21, *P* = 0.56). The results indicate that Mutect2 has missed a large fraction of CHIP mutations and that VarTracker could reliably identify CHIP mutations with high sensitivity. Also, the refinement strategy could rescue true CHIP mutations. With these key features, UNISOM could enhance the detection of associations between CHIP mutations and important clinical variables, even with a modest cohort size.

In summary, by applying the pipeline to the Mayo Biobank WGS data, our analyses revealed similar trends, as previously discovered in much large cohorts [4,7,8], in terms of most commonly mutated genes, type of mutations, and mutation signature, as well as CHIP association with age and smoking status. In particular, the meta-calling approach markedly enhances the detection of low-VAF mutations, revealing high CHIP prevalence in this cohort with ∼ 30× coverage.

## Discussion

In the general population without evidence of hematologic disorders, CHIP prevalence is estimated at ∼ 3%–5% based on WES [7,8,82] and WGS [4] data. However, limited by the coverage, sequencing error (> 0.1%) [83], and the power of analysis pipeline, WGS and WES likely miss a high proportion of small mutant clones [5]. To support this, smMIPs sequencing, which has an error rate of 10-fold lower compared to conventional sequencing, recovered over half of the CHIP mutations missed by WGS data with ∼ 30× coverage [17]. To tackle the analytical challenge, we present UNISOM as a standalone application for CHIP prediction. UNISOM utilizes a meta-calling strategy to achieve high sensitivity in variant detection, combined with a ML-based model for variant classification.

Applied to the Mayo Biobank WGS data, we revealed a CHIP prevalence of 17.5% that is much higher compared to previous studies using a single caller [4,7], which is less sensitive to low-VAF mutations. Notably, over half of the clones carrying CHIP mutations continue to grow [14,17]. For example, samples taken from individuals ∼ 6 years before diagnosed with AML were found to accumulate more mutations and show greater clonal expansion in peripheral blood compared to the control [14]. Thus, implementing sensitive tools like UNISOM will improve the early detection of low-VAF CHIP mutations prior to diagnosis, enabling monitoring of the carriers and possible intervention [13,82].

Furthermore, analysis of longitudinal data began to shed insight into the dynamics of small clones [84]. Variant fitness effect model predicted that over 80% of the variants with ≥ 1% VAF continue expansion over time [85]. Consequently, some of the growing clones with ≥ 1% VAF, initially not considered as CHIP, will reach ≥ 2% VAF years later [17,85]. It has been reported that clonal mutations with ≥ 1% VAF also confer an increased risk of developing AML [6]. Although clinical implications of small clones remain unclear [5], the possibility of their expansion into larger clones suggests that close monitoring should be considered. Currently, UNISOM is limited to the detection of somatic mutations in leukemia driver genes. The applicability of UNISOM to other genes requires further testing.

UNISOM is sensitive even at insufficient sequencing coverage, as in the case of Mayo Biobank WGS data. UNISOM identified 30% of the CHIP mutations with 2%–5% VAFs and another 54% with 5%–10% VAFs, at a lenient cutoff of ≥ 2 supporting reads. The power of our approach is well illustrated by the CHIP mutations identified in *TP53* (Figure 5D). Not just in cancers [86], *TP53* mutations are also prevalent in blood cells of healthy individuals [87]. Based on error-corrected targeted sequencing data, about half of the cancer-free elderly individuals carry rare TP53 mutations with 0.01%–0.37% VAFs [87]. A similar phenomenon was also observed in patients with ovarian cancer and without cancer, who carried mutations with 0.01%–0.1% VAFs that were predicted as being likely functional [88]. Thus, low-VAF somatic mutations in *TP53* are unlikely to be noise. In the Mayo Biobank, *TP53* is the third most frequently mutated gene, with a total of 15 (10 known) genomic positions carrying CHIP mutations, at 3.1%–8.0% VAFs. All these mutations were present in exons 5–9, consistent with the report that *TP53* mutations are predominantly clustered in exons 4–10 [88]. Based on targeted error-corrected sequencing [6,9,14,87,89] or error-corrected duplex sequencing [20,90], which are sensitive to low-VAF mutations, 10 (7 known and 3 novel) of the 15 positions were found to carry CH mutations. All the 10 positions with known CHIP mutations in Mayo Biobank overlapped those identified in the TOPMed WGS data (∼ 40×) [4], but the VAFs were 2.3- to 5.1-fold lower in the former. Not surprisingly, none of the *TP53* mutations in Mayo Biobank were detected by Mutect2 that was found to be highly insensitive on 40× WGS data [4].

Our study is limited by the relatively small cohort size in Mayo Biobank, as compared to the WGS-based CHIP study in TOPMed cohort. Consequently, we failed to detect some of the known CHIP mutations, such as those located in mutation hotspots in splicing factor genes *SRSF2* and *U2AF1*.

## Code availability

The UNISOM pipeline is freely available at GitHub (https://github.com/shulanmayo/UNISOM). The code has also been submitted to BioCode at the National Genomics Data Center (NGDC), China National Center for Bioinformation (CNCB) (BioCode: BT007816), which is publicly accessible at https://ngdc.cncb.ac.cn/biocode/tool/7816.

## CRediT author statement

**Shulan Tian:** Conceptualization, Project administration, Supervision, Writing – original draft, Writing – review & editing. **Garrett Jenkinson:** Methodology, Software, Writing – original draft, Writing – review & editing. **Alejandro Ferrer:** Resources, Writing – review & editing. **Huihuang Yan:** Formal analysis, Writing – review & editing. **Joel A. Morales-Rosado:** Resources, Writing – review & editing. **Kevin L. Wang:** Formal analysis. **Terra L. Lasho:** Resources, Writing – review & editing. **Benjamin B. Yan:** Formal analysis. **Saurabh Baheti:** Methodology, Investigation. **Janet E. Olson:** Resources, Writing – review & editing. **Linda B. Baughn:** Investigation, Writing – review & editing. **Wei Ding:** Resources, Writing – review & editing. **Susan L. Slager:** Methodology, Writing – review & editing. **Mrinal S. Patnaik:** Methodology, Writing – review & editing. **Konstantinos N. Lazaridis:** Resources, Writing – review & editing. **Eric W. Klee:** Conceptualization, Funding acquisition, Project administration, Supervision, Writing – original draft, Writing – review & editing. All authors have read and approved the final manuscript.

## Competing interests

The authors have declared no competing interests.

## Supplementary material

Supplementary material is available at *Genomics, Proteomics & Bioinformatics* online (https://doi.org/10.1093/gpbjnl/qzaf040).

## Supplementary Material

qzaf040_Supplementary_Data

## ORCID

0000-0002-3348-7439 (Shulan Tian)

0000-0003-2548-098X (Garrett Jenkinson)

0000-0002-4883-3023 (Alejandro Ferrer)

0000-0003-0756-2922 (Huihuang Yan)

0000-0002-7710-2211 (Joel A. Morales-Rosado)

0009-0004-4602-8065 (Kevin L. Wang)

0000-0002-0315-5230 (Terra L. Lasho)

0009-0002-4373-1279 (Benjamin B. Yan)

0000-0002-3564-7627 (Saurabh Baheti)

0000-0003-4944-7789 (Janet E. Olson)

0000-0001-5229-4897 (Linda B. Baughn)

0000-0001-9811-4601 (Wei Ding)

0000-0002-5173-4712 (Susan L. Slager)

0000-0001-6998-662X (Mrinal S. Patnaik)

0000-0002-0437-681X (Konstantinos N. Lazaridis)

0000-0003-2946-5795 (Eric W. Klee)
