# Supplementary material for: UNISOM: Unified Somatic Calling and Machine Learning-based Classification Enhance the Discovery of CHIP
Source: Genomics Proteomics Bioinformatics. 2025 Apr 29;23(2):qzaf040. doi: 10.1093/gpbjnl/qzaf040 (PMC12282763; doi:10.1093/gpbjnl/qzaf040)
Supplement: qzaf040_Supplementary_Data [file qzaf040_supplementary_data.zip › Figure S1.pptx]

## Slide 1
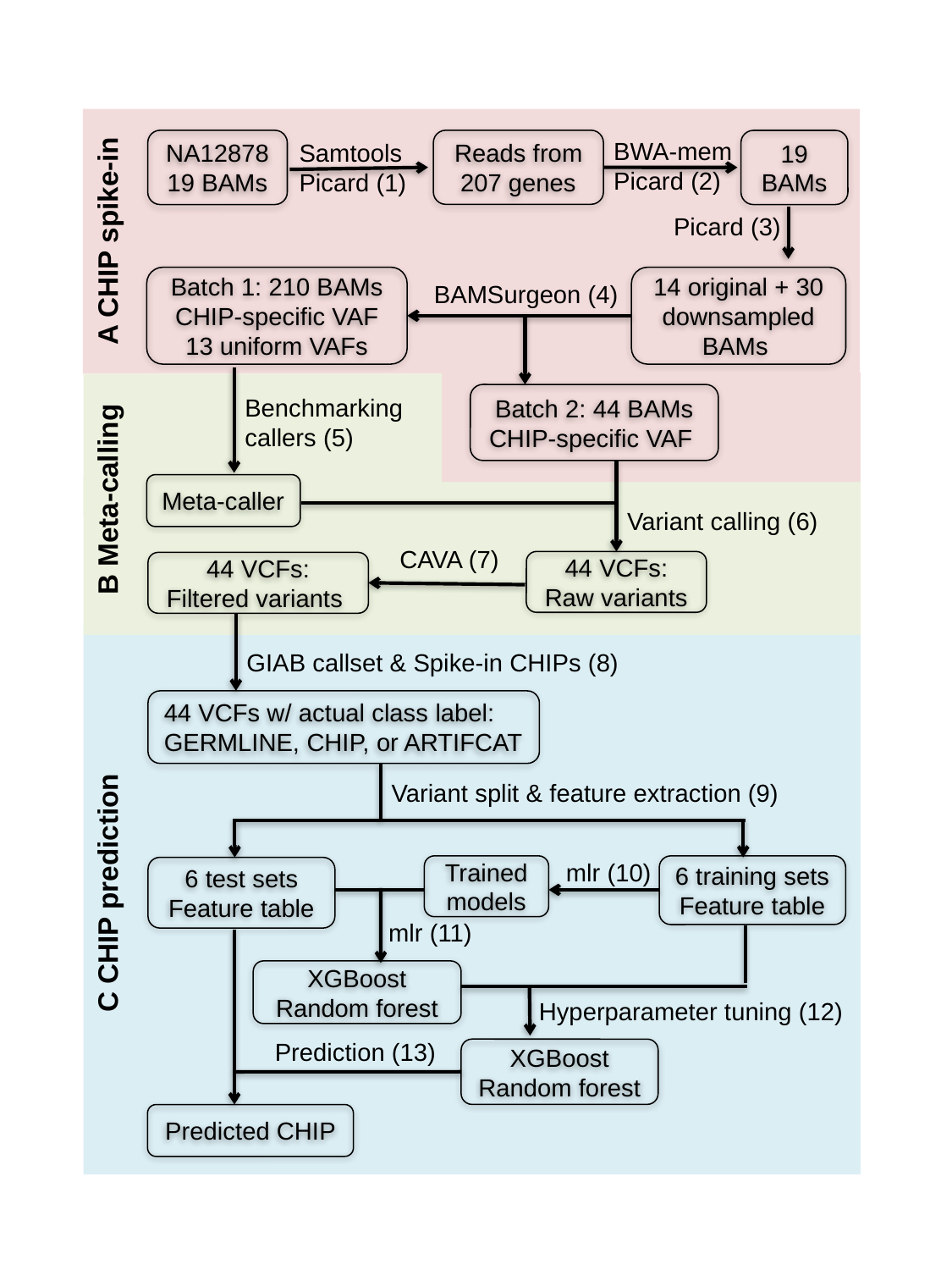

BWA-mem
Picard (2)
NA12878
19 BAMs
Reads from 207 genes
19 BAMs
Samtools
Picard (1)
Picard (3)
A CHIP spike-in
Batch 1: 210 BAMs
CHIP-specific VAF
13 uniform VAFs
14 original + 30 downsampled BAMs
BAMSurgeon (4)
Batch 2: 44 BAMs
CHIP-specific VAF
Benchmarking
callers (5)
Meta-caller
B Meta-calling
Variant calling (6)
CAVA (7)
44 VCFs: Raw variants
44 VCFs: Filtered variants
GIAB callset & Spike-in CHIPs (8)
44 VCFs w/ actual class label: GERMLINE, CHIP, or ARTIFCAT
Variant split & feature extraction (9)
mlr (10)
Trained models
6 training sets
Feature table
6 test sets
Feature table
C CHIP prediction
mlr (11)
XGBoost
Random forest
Hyperparameter tuning (12)
Prediction (13)
XGBoost
Random forest
Predicted CHIP
