# Supplementary material for: UNISOM: Unified Somatic Calling and Machine Learning-based Classification Enhance the Discovery of CHIP
Source: Genomics Proteomics Bioinformatics. 2025 Apr 29;23(2):qzaf040. doi: 10.1093/gpbjnl/qzaf040 (PMC12282763; doi:10.1093/gpbjnl/qzaf040)
Supplement: qzaf040_Supplementary_Data [file qzaf040_supplementary_data.zip › Figure S3.pdf]

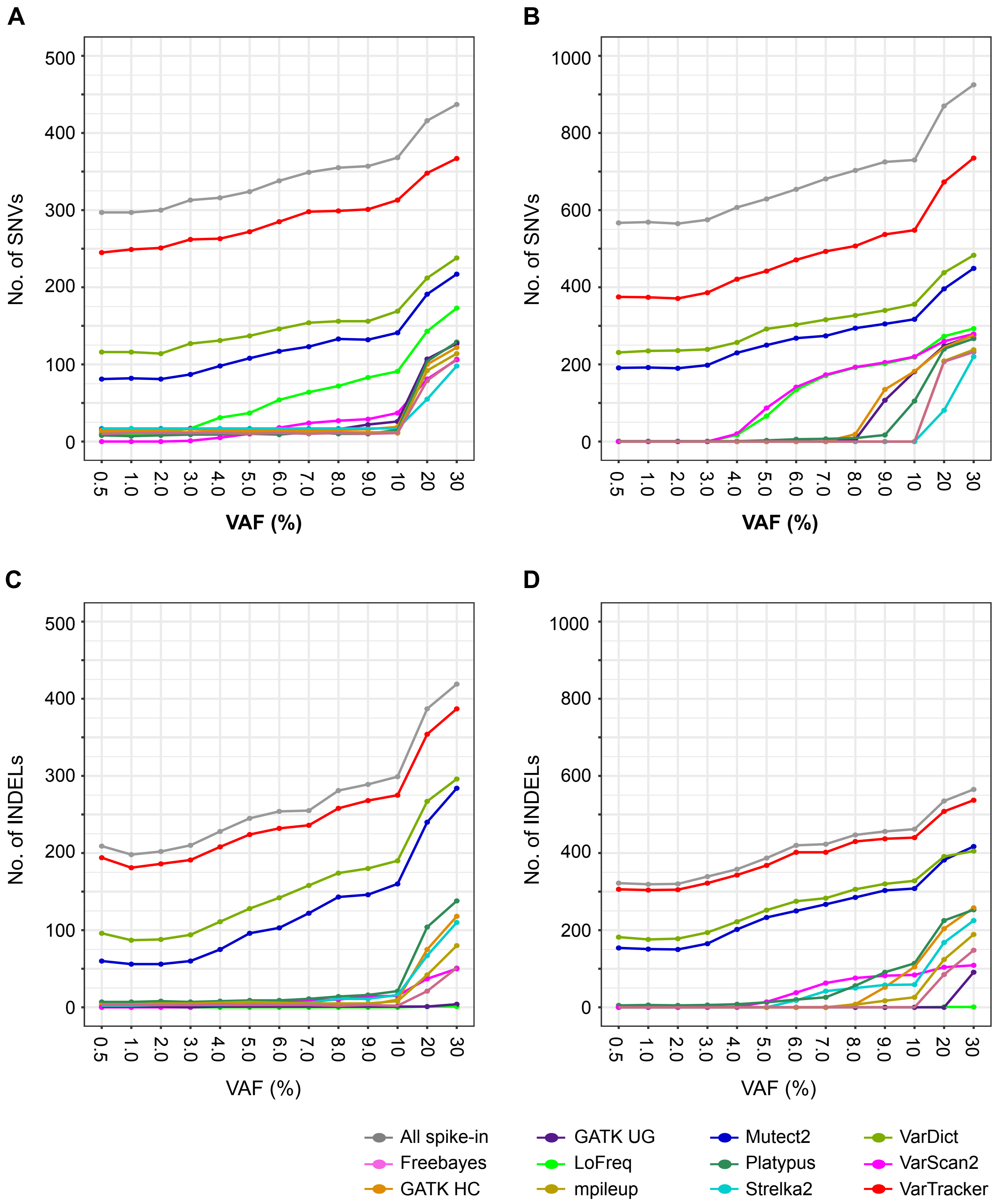
A
B
C
D
No. of SNVs
No. of INDELs
VAF (%)
0
100
200
300
400
500
200
400
600
800
1000
0.5
1.0
2.0
3.0
4.0
5.0
6.0
7.0
8.0
9.0
10
20
30
All spike-in
Freebayes
GATK HC
GATK UG
LoFreq
mpileup
Mutect2
Platypus
Strelka2
VarDict
VarScan2
VarTracker
