# Supplementary material for: UNISOM: Unified Somatic Calling and Machine Learning-based Classification Enhance the Discovery of CHIP
Source: Genomics Proteomics Bioinformatics. 2025 Apr 29;23(2):qzaf040. doi: 10.1093/gpbjnl/qzaf040 (PMC12282763; doi:10.1093/gpbjnl/qzaf040)
Supplement: qzaf040_Supplementary_Data [file qzaf040_supplementary_data.zip › File S1.docx]

**File S1 Supplementary methods and note 1**

### **Known CHIP mutations**

A list of 2367 CHIP mutations, including 1331 SNVs and 1036 INDELs (Table S1), was compiled from 2 WES-based CHIP studies [1,2] and the WGS-based Trans-Omics for Precision Medicine (TOPMed) study [3]. The analysis was based on the following rules: (1) SNVs were prioritized over INDELs if both were reported at the same genomic position across these studies or across different subjects in the same study; (2) for variants identified at the same genomic position in multiple subjects, the one with the lowest variant allele frequency (VAF) was retained along with its alternate base in that subject; (3) for a given variant, if two or three alternate bases were identified at the same VAF, one of them was randomly selected; and (4) INDELs were normalized as left-aligned and parsimonious with GATK walker LeftAlignAndTrimVariants. In the resulting list, over 92% of the CHIP mutations were identified from WGS in the TOPMed cohort that has the largest sample size with nearly 97,700 participants [3].

**ML-based variant classifier**

CHIP mutations often had low VAFs, in the same range as artifacts from DNA contamination and damage, or from sequencing and alignment errors, which makes VAF-based filtering problematic. Also, due to the much lower somatic mutation rates in normal cells, artifacts are often observed much more frequently than true somatic variants. Thus, the filtering process is very critical to refine true CHIP mutations but is poorly standardized and time-consuming.

The traditional hard-filtering approach is based on attributes from variant annotations, such as sequence context and the number of reads supporting alternative allele. It performs a multiple-step filtering based on the pre-defined thresholds of individual attributes. With the challenges of setting optimal thresholds across all selected attributes simultaneously, this approach will filter out true mutations if they fail to meet any of the thresholds. In addition, CHIP mutations have characteristic mutational signatures [1,3], which probabilistically contribute to CHIP prediction but are difficult to be incorporated into the hard-filtering approach. On the other hand, machine learning (ML) based models learn information from annotation profiles in the training set that can be used to discriminate real somatic mutations (CHIP) from germline variants and artifacts. Based on the assessment made on the test set, the best-performing ML model(s) will be selected and further applied to the real data for CHIP prediction. Unlike hard-filtering that requires multiple rounds of selections, ML-based probabilistic classifiers enable one-time binary selection of the predicted CHIP mutations versus non-CHIP noise. Thus, to streamline the CHIP detection, we developed ML-based prediction models based on variant-associated features.

To build ML-based prediction models, raw variants in NA12878 were pre-assigned with class labels, as described below, split into SNVs and INDELs, and the associated features were extracted. Four algorithms were each tested on SNVs and INDELs called from WES, WGS and the combined data (i.e., 6 tests per algorithm), at default parameter setting. In addition, neural network was tested on WGS data. In calculating the performance metrics, the pre-assigned class labels were used to represent the actual class labels. Based on the performance metrics, XGBoost and Random forest were selected for further hyperparameter tuning. Key steps are illustrated in Figure S1 and described below in detail.

**Class labels**

Variants from meta-calling in NA12878 were each assigned with an actual class label of being CHIP, GERMLINE, or ARTIFACT. To identify those that are most likely germline variants, we used a set of SNVs and short INDELs with high-confidence in NA12878 (NISTv3.3.2) as the ground truths [4], which is generated by the Genome in a Bottle (GIAB) Consortium and publicly available at <https://ftp>-trace.ncbi.nlm.nih.gov/ReferenceSamples/giab/release/NA12878_HG001/latest/GRCh37/SupplementaryFiles/[HG001_GRCh37_1_22_v4.2.1_all.vcf.gz](https://ftp-trace.ncbi.nlm.nih.gov/ReferenceSamples/giab/release/NA12878_HG001/latest/GRCh37/SupplementaryFiles/HG001_GRCh37_1_22_v4.2.1_all.vcf.gz). A variant is flagged as GERMLINE if (1) it overlaps the above GIAB list of known germline variants; or (2) it is a novel one but identified by at least two of the callers, with > 30% VAF, the VAF cutoff of being germline as previously suggested [2,5]. The second criterion for including novel ones considers a possibility that the GIAB list may still miss some germline variants. On the other hand, a variant is flagged as CHIP if (1) it is mapped to the genomic position carrying a CHIP spike-in; or (2) it is called from elsewhere by at least two of the callers with $\leq$30% VAF, considering that NA12878 itself may also carry CHIP. The remaining variants are labeled as ARTIFACT. They were used as the actual class labels in calculating performance metrics on the test set for CHIP prediction models.

**Feature extraction**

To build a well-performing variant classifier, it is critical to select the features that together can potentially characterize a raw variant, so that the algorithm can learn the underlying discriminatory patterns differentiating between CHIP, GERMLINE, and ARTIFACT. For each raw SNV and INDEL with CAVA predicted functional effects, we collected 25 and 23 features, respectively, that describe meta-calling status, variant quality metrics, the associated genomic context, as well as the status in five public variant databases (Table S4**)**. Additionally, we included mutational signature, considering that, in CH, > 50% of the observed point mutations are C-to-T transitions [1,2], which are the dominant point mutations in most aging tissues [1,6]. GATK walkers VariantAnnotator and VariantFiltration were used to extract these features.

**Model training**

Given the raw variants in the feature space, variant classifier predicts which ones are real CHIP with functional effects. As calling accuracy of INDEL remains lower than that of SNV [7], the two were separated in model training. Also, considering the possible differences in sequencing platforms, variant classifier was built separately for WES, WGS and the combined data. To avoid overfitting, for each of the six callsets (2 variant types x 3 data types), we applied the holdout method by randomly selecting 80% of the raw variants as the training set and leaving the remaining 20% as the test set.

To start model training, all 11 categorical variables were one-hot encoded into (Table S4**)**. For each of the six callsets, the mlr R package [8] was used to build four ML models, including Recursive Partitioning and Regression Trees (rpart), Random forest, Support Vector Machine (SVM), and eXtreme Gradient Boosting (XGBoost). We ran the 4 models on the training set, with the default parameters, and evaluated their performance in class prediction.

We also tested neural network in CHIP prediction, using WGS data from batch 2 simulation with CHIP-specific VAFs. It took the same 26 (for SNV) and 24 (for INDEL) features used to train the 4 ML-based models described above. Neural network model was built with TensorFlow platform (<https://www.tensorflow.org>) in python environment.

**Hyperparameter tuning**

XGBoost and Random forest based classifiers clearly demonstrated better performance in predicting CHIP compared to the other models. Thus, both were selected for hyperparameter tuning on the training set, aiming to optimize the model performance by adjusting various hyperparameters. Hyperparameter tuning used k-fold cross-validation (here k = 5), which was compared to the model built with default parameters.

**Confidence interval estimation**

To facilitate the prioritization**,** following the **above prediction refinement,** a hierarchical Bayesian model was developed t**o estimate** confidence intervals for CHIP predictions. The model utilizes CHIP incidence in a reference population of healthy individuals as the background. It is known that CHIP mutations are very rare in young individuals under the age 40 [1,2]. In numerous studies, healthy individuals in this age range were selected as normal controls when calling CHIP mutations from WGS [3] and WES data [9]. To build the reference, **WES data (median coverage 125X) were generated from** 75 healthy individuals younger than 40 years of age. Under the model, CHIP is defined as the allele deviated from the reference one in this healthy population. Then, in an individual, the probability of detecting CHIP at a given position can be estimated based on the background model (File S2).

**CHIP prediction with WES data**

The performance of meta-caller and XGBoost classifier was further evaluated on real WES data from a cohort of 25 individuals, for whom targeted deep sequencing data were also available. The study was approved by the institutional review board at Mayo Clinic (Mayo Clinic IRB 16-004173). Peripheral blood mononuclear cells were collected, with written consent, from individuals who were examined by a medical professional for the absence of cytopenias, multiple myeloma and other hematologic disorders in blood. Bone marrow was also examined to verify the absence of multiple myeloma and lymphoma. DNA isolation, WES library construction, Illumina paired-end sequencing, reads mapping and post-alignment processing were performed as previously described [10]. WES had a median coverage of 158X (127–179X), estimated by the GATK walker DepthOfCoverage. Variants were detected with meta-caller, with default parameter setting, followed by functional annotation with CAVA. Those predicted to have functional effects were used as input for the XGBoost classifier pretrained on simulated WES data.

To assess the CHIP predictions on WES made by the XGBoost classifier, we used CHIP variants identified from targeted sequencing data as ground truths. In brief, DNA from the same 25 individuals with WES data was deeply sequenced on a customized panel of 189 leukemia-associated genes (Table S1). Library construction, paired-end sequencing and bioinformatics analysis were as described [11]. The 25 samples have approximately 95% of the targeted regions sequenced at $\geq$100X coverage, with ~ 50% up to 3000–4000X coverage and a median coverage of approximately 1000X. At the aforementioned sequencing coverage, variants with > 0.5% VAF should be identified with high confidence. Candidate mutations were identified by two somatic calling pipelines, an in-house pipeline using BWA-mem [12] and GATK HaplotypeCaller and the commercial Agilent SureCall (v4.1.2, BWA and SNPPET SNV caller), following the criteria used in [11]. Variants were filtered out if present in any of the four public germline databases listed in Table S4 (the 1000 Genomes Project, gnomAD, ExAC, and TOPMed), with $\geq$0.3% minor allele frequency (MAF).

The retained variants were classified as CHIP if they met any of the following criteria: (1) overlap with CHIP-associated driver mutations defined in [13]; (2) present in catalogue of somatic mutations in cancer (COSMIC) database; (3) predicted as being deleterious by SIFT [14], MutationTaster2 [15], or Polyphen-2 [16]; or (4) with functional relevance in leukemia based on literature search. Finally, the identified CHIP mutations were manually inspected with Alamut Visual software (v2.7, Interactive Biosoftware, Rouen, Haute-Normandie, France), by which potential sequencing artifacts were filtered out. We only kept those in the list of known driver mutations.

After the above manual curation, 45 CHIP mutations were retained. Those mapped outside of the WES capture regions (5 mutations) or with no coverage in WES (7 mutations) were filtered out. The remaining 33 mutations (45 – 5 – 7) were used as ground truths when assessing XGBoost predictions on WES from the same cohort. We used recall, based on formula (2), to measure the predictive performance, where true positive and false negative are those from the 33 mutations that are detected and missed by UNISOM, respectively.

**References**

[1] Jaiswal S, Fontanillas P, Flannick J, Manning A, Grauman PV, Mar BG, et al. Age-related clonal hematopoiesis associated with adverse outcomes. N Engl J Med 2014;371:2488–98.

[2] Genovese G, Kahler AK, Handsaker RE, Lindberg J, Rose SA, Bakhoum SF, et al. Clonal hematopoiesis and blood-cancer risk inferred from blood DNA sequence. N Engl J Med 2014;371:2477–87.

[3] Bick AG, Weinstock JS, Nandakumar SK, Fulco CP, Bao EL, Zekavat SM, et al. Inherited causes of clonal haematopoiesis in 97,691 whole genomes. Nature 2020;586:763–8.

[4] Zook JM, McDaniel J, Olson ND, Wagner J, Parikh H, Heaton H, et al. An open resource for accurately benchmarking small variant and reference calls. Nat Biotechnol 2019;37:561–6.

[5] Kraft IL, Godley LA. Identifying potential germline variants from sequencing hematopoietic malignancies. Blood 2020;136:2498–506.

[6] Alexandrov LB, Nik-Zainal S, Wedge DC, Aparicio SAJR, Behjati S, Biankin AV, et al. Signatures of mutational processes in human cancer. Nature 2013;500:415–21.

[7] Ghoneim DH, Myers JR, Tuttle E, Paciorkowski AR. Comparison of insertion/deletion calling algorithms on human next-generation sequencing data. BMC Res Notes 2014;7:864.

[8] Bischl B, Lang M, Kotthoff L, Schiffner J, Richter J, Studerus E, et al. mlr: Machine Learning in R. J Mach Learn Res 2016;17:1–5.

[9] Niroula A, Sekar A, Murakami MA, Trinder M, Agrawal M, Wong WJ, et al. Distinction of lymphoid and myeloid clonal hematopoiesis. Nat Med 2021;27:1921–7.

[10] Mangaonkar AA, Ferrer A, Pinto EVF, Cousin MA, Kuisle RJ, Gangat N, et al. Clinical Applications and Utility of a Precision Medicine Approach for Patients With Unexplained Cytopenias. Mayo Clin Proc 2019;94:1753–68.

[11] Kusne Y, Lasho T, Mangaonkar A, Tefferi A, Gangat N, Finke C, et al. Remarkable stability in clonal hematopoiesis involving leukemia-driver genes in patients without underlying myeloid neoplasms. Am J Hematol 2021;96:E392-e6.

[12] Li H. Aligning sequence reads, clone sequences and assembly contigs with BWA-MEM. arXiv:1303.3997 2013.

[13] Mouhieddine TH, Sperling AS, Redd R, Park J, Leventhal M, Gibson CJ, et al. Clonal hematopoiesis is associated with adverse outcomes in multiple myeloma patients undergoing transplant. Nat Commun 2020;11:2996.

[14] Ng PC, Henikoff S. SIFT: Predicting amino acid changes that affect protein function. Nucleic Acids Res 2003;31:3812–4.

[15] Schwarz JM, Cooper DN, Schuelke M, Seelow D. MutationTaster2: mutation prediction for the deep-sequencing age. Nat Methods 2014;11:361–2.

[16] Adzhubei I, Jordan DM, Sunyaev SR. Predicting functional effect of human missense mutations using PolyPhen-2. Curr Protoc Hum Genet 2013;Chapter 7:Unit7.20.
