# Supplementary material for: UNISOM: Unified Somatic Calling and Machine Learning-based Classification Enhance the Discovery of CHIP
Source: Genomics Proteomics Bioinformatics. 2025 Apr 29;23(2):qzaf040. doi: 10.1093/gpbjnl/qzaf040 (PMC12282763; doi:10.1093/gpbjnl/qzaf040)
Supplement: qzaf040_Supplementary_Data [file qzaf040_supplementary_data.zip › supplementary material captions.docx]

**Supplementary material**

**File S1 Supplementary methods and note 1**

**File S2 Supplementary note 2**

**Figure S1** **Flowchart for developing the meta-caller and variant classifier**

**A.** CHIP spike-in (step 1–4). NA12878 WES and WGS alignments within the 202 leukemia-associated genes were extracted, remapped to the hg19 genome reference, and then downsampled to a minimum coverage of 20X (Table S2). Of the resulting 44 BAMs, 21 were used as inputs in batch 1 simulation, as shown in Table S2. The 2367 known CHIP were added as spike-in at CHIP-specific VAFs, or at each of 13 uniform VAFs between 0.5% and 30%. Batch 2 simulation was performed on all 44 BAMs using CHIP-specific VAFs. **B.** Variant detection using meta-caller (step 5–7). Batch 1 simulated data were used to benchmark 10 open source tools plus VarTracker. The meta-caller was built by combining VarTracker with Mutect2 and Vardict, the 2 top-performing tools. Variants were called from batch 2 simulation data and used to develop the variant classifier. **C.** CHIP prediction (step 8–13). Raw variants with functional effects (via CAVA annotation) were each assigned an actual class label of GERMLINE, CHIP, or ARTIFCAT; the associated features were extracted from VCF (Table S4). The annotated variants were then used as the training sets (80% of the data) to build machine learning-based prediction models in the feature space, separately for variant type (SNV and INDEL) and sequencing type (WES, WGS, and both combined). XGBoost performed well (with the highest F1-score) on predicting CHIP in the test sets (20% of the data), followed by Random forest. Both were selected for hyperparameter tuning, and used to predict CHIP in the test sets. Numbers in the parenthesis indicate analysis steps in order. CAVA, clinical annotation of variants; CHIP, clonal hematopoiesis of indeterminate potential; XGBoost, eXtreme Gradient Boosting; GIAB, Genome in a Bottle; SNV, single nucleotide variant; VAF, variant allele frequency; VCF, variant call format; WES, whole-exome sequencing; WGS, whole-genome sequencing.

**Figure S2** **VAF distribution for known and simulated CHIP mutations**

For simulated data with CHIP-specific VAFs, VAFs were estimated based on read pileup at sites carrying pre-inserted mutations. WES, WES_NA12878_01 simulated at 50X; WGS, WGS_NA12878_01 simulated at 50X; Observed, VAFs from the 2367 known CHIP (1331 SNVs and 1036 INDELs). In both simulated WES and WGS, the spike-in CHIP had reduced VAFs compared to the known CHIP.

**Figure S3** **Recovery of simulated CHIP varies by callers over different VAFs**

**A.** and **C.** SNVs and INDELs simulated at 100X in NA12878_01 WES data. **B.** and **D.** simulated at 100X in NA12878_01 WGS data. Data are from batch 1 simulation. BAMSurgeon was used to spike-in known SNVs/INDELs at each of the 13 uniform VAFs between 0.5% and 30% (X-axis). Y-axis shows the number of total spike-in (gray line) and the number of spike-in that were recovered by individual callers (color lines). VarTracker, followed by VarDict and GATK Mutect2, identified the largest number of spiked-in, particularly at low VAFs. See Table S2 for details about the 2 NA12878 samples. GATK HC, GATK HaplotypeCaller; GATK UG, GATK UnifiedGenotyper.

**Figure S4** **Precision of 11 tools benchmarked on simulated data**

**A.** Simulated SNVs and INDELs in WES data. **B.** Simulated SNVs and INDELs in WGS data. Each plot in (A) and (B) used precision estimated from 7 WES and 11 WGS data, respectively, split into SNV and INDEL. All data are from batch 1 simulation (Table S2) that used CHIP-specific VAFs, excluding those with > 100X coverage.

**Figure S5 Recovery of CHIP simulated at different coverage**

**A.** NA12878_01 WES. **B.** NA12878_02 WES. **C.** NA12878_01 WGS. **D.** NA12878_02 WGS. Y-axis shows the number of SNVs and INDELs that were spiked-in (gray lines) and recovered by the 3 callers (color lines). Overall, the numbers increase following the coverage increase for both WES and WGS. Data are from batch 1 simulation using CHIP-specific VAFs. See Table S2 about the WES and WGS data used on simulation.

**Figure S6 Performance metrics before and after hyperparameter tuning**

**A.** Recall rate of CHIP prediction based on XGBoost and random forest. **B.** Precision. **C.** Accuracy. Recall, precision, and accuracy were estimated using formula (2), (1), and (5), respectively. Data were from batch 2 simulation that used CHIP-specific VAFs. Raw variants were called from BAMs by meta-caller, annotated, filtered, and assigned with actual labels of CHIP, GERMLINE, or ARTIFACT.

**Figure S7** **Performance metrics of machine learning algorithms in predicting germline variants**

**A.** Recall of SNV prediction. **B.** Recall of INDEL prediction. **C.** Precision of SNV prediction. **D.** Precision of INDEL prediction. **E.** Accuracy of SNV prediction. **F.** Accuracy of INDEL prediction. The prediction models were applied to the lists of CAVA-annotated variants called from WES (25 BAMs), WGS (19 BAMs), and both (44 BAMs) with the Meta-caller, separated into SNVs and INDELs. Each variant in the input VCF was assigned with an actual class label of “GERMLINE”, “CHIP”, or “ARTIFCAT”, described in detail in “Class labels” in File S1. Individual steps in simulation (step 1–4), meta-calling (step 6–7), and variant classification (step 8–13) are illustrated in Figure S1. Data are from batch 2 simulation using CHIP-specific VAFs. See Table S2 for more information about the WES and WGS data. SVM, Support Vector Machine.

**Figure S8** **Plot of individuals with two or more mutations**

Columns represent the 28 individuals carrying co-mutation. Ten of the genes (in bold) were found to show co-mutation in a previous study (PMID: 25426837).

**Table S1** **Collection of leukemia-associated genes and known CHIP mutations**

**Table S2 NA12878 WGS and WES data used in this study**

**Table S3 Eleven tools benchmarked for single-sample variant detection**

**Table S4** **Features used to build ML variant classifier**

**Table S5 Performance metrics of neural network in CHIP prediction**

**Table S6 Predicted CHIP mutations in Mayo Biobank**
