# Supplementary material for: UNISOM: Unified Somatic Calling and Machine Learning-based Classification Enhance the Discovery of CHIP
Source: Genomics Proteomics Bioinformatics. 2025 Apr 29;23(2):qzaf040. doi: 10.1093/gpbjnl/qzaf040 (PMC12282763; doi:10.1093/gpbjnl/qzaf040)
Supplement: qzaf040_Supplementary_Data [file qzaf040_supplementary_data.zip › Table S1.docx]

**Table S1** **Collection of leukemia-associated genes and known CHIP mutations**

| **Data** | **Type** | **No. of gene** | **No. of SNV** | **No. of INDEL** | **No. of subject** | **Source** |
| --- | --- | --- | --- | --- | --- | --- |
| 1 | WES | 156 | 310 | 181 | 17,182 | PMID: 25426837 |
| 2 | WES | 14 | 144 | 68 | 12,380 | PMID: 25426838 |
| 3 | WES | 31 | 65 | 12 | 2728 | PMID: 25326804 |
| 4 | WGS | 74 | 1168 | 1082 | 97,691 | PMID: 33057201 |
| 5 | NA | 189 | NA | NA | NA | Mayo Clinic CHIP panel |
| Total |  | 202 | 1331 | 1036 |  |  |

*Note*: Genomic positions are not available for the 77 CHIP mutations (65 SNVs and 12 INDELs) identified in PMID: 25326804. Mayo Clinic CHIP panel only has the gene list without variant information. CHIP, clonal hematopoiesis of indeterminate potential; INDEL, insertion or deletion; SNV, single nucleotide variant; WES, whole-exome sequencing; WGS, whole-genome sequencing.
