# Supplementary material for: UNISOM: Unified Somatic Calling and Machine Learning-based Classification Enhance the Discovery of CHIP
Source: Genomics Proteomics Bioinformatics. 2025 Apr 29;23(2):qzaf040. doi: 10.1093/gpbjnl/qzaf040 (PMC12282763; doi:10.1093/gpbjnl/qzaf040)
Supplement: qzaf040_Supplementary_Data [file qzaf040_supplementary_data.zip › Table S2.docx]

**Table S2** **NA12878 WGS and WES data used in this study**

| **Data** | **Type** | **Platform** | **Sample ID** | **Length (bp)** | **Original coverage (X)** | **Coverage used (X)** | **Source** |
| --- | --- | --- | --- | --- | --- | --- | --- |
| 1 | WGS | HiSeq 2500 | GIAB_HG001_WGS | 148 | 300 | 200, 100, 50, 20 | GIAB (PMID: 30936564) |
| 2 | WGS | HiSeq 4000 | SRR8454587 | 150 | 29 | 29 | SRA (PMID: 31249349) |
| 3 | WGS | HiSeq 4000 | SRR8454588 | 150 | 29 | 29 | SRA (PMID: 31249349) |
| 4 | WGS | NovaSeq 6000 | SRR8454589 | 150 | 29 | 29 | SRA (PMID: 31249349) |
| 5 | WGS | HiSeq X Ten | SRR6885087 | 150 | 37 | 37 | SRA |
| 6 | WGS | HiSeq X Ten | SRR7733437 | 150 | 24 | 24 | SRA |
| 7 | WGS | HiSeq X Ten | SRR7781427 | 150 | 34 | 34 | SRA |
| 8 | WGS | HiSeq X Ten | SRR7781429 | 150 | 40 | 40 | SRA |
| 9 | WGS | HiSeq X Ten | SRR7781444 | 150 | 37 | 37 | SRA (PMID: 31249349) |
| 10 | WGS | HiSeq X Ten | SRR7781431 | 150 | 24 | 24 | SRA (PMID: 31249349) |
| 11 | WGS | NovaSeq | NA12878_01_WGS | 150 | 100 | 100, 50, 20 | Internal data |
| 12 | WGS | NovaSeq | NA12878_02_WGS | 150 | 84 | 84, 50, 20 | Internal data |
| 13 | WES | HiSeq 2500 | GIAB_HG001_WES | 100 | 100 | 100 | GIAB (PMID: 30936564) |
| 14 | WES | HiSeq 4000 | SRR8381428 | 148 | 355 | 200, 100, 50, 20 | SRA |
| 15 | WES | NovaSeq 6000 | SRR8381429 | 145 | 213 | 200, 100, 50, 20 | SRA (PMID: 31249349) |
| 16 | WES | HiSeq 4000 | ERR1905889 | 150 | 318 | 200, 100, 50, 20 | SRA |
| 17 | WES | HiSeq 4000 | ERR1905890 | 150 | 360 | 200, 100, 50, 20 | SRA |
| 18 | WES | HiSeq 2500 | NA12878_01_WES | 102 | 132 | 132, 100, 50, 20 | Internal data |
| 19 | WES | HiSeq 2500 | NA12878_02_WES | 102 | 135 | 135, 100, 50, 20 | Internal data |

*Note*: Sequence at SRA can be downloaded through the web link: https://trace.ncbi.nlm.nih.gov/Traces/sra/?run=accession, with accession listed the "Sample ID" column. Datasets 5 and 6 were generated from PCR-free libraries. Batch 1 simulation used 21 BAMs from 5 WGS (1, 3, 4, 11, and 12) and 3 WES data (13, 18, and 19), with 13 uniform and also CHIP-specific VAFs. Batch 2 simulation used all 44 BAMs from the 19 datasets, with CHIP-specific VAFs. GIAB, Genome in a Bottle; PCR, polymerase chain reaction; SRA, Sequence Read Archive; VAF, variant allele frequency.
