# Supplementary material for: UNISOM: Unified Somatic Calling and Machine Learning-based Classification Enhance the Discovery of CHIP
Source: Genomics Proteomics Bioinformatics. 2025 Apr 29;23(2):qzaf040. doi: 10.1093/gpbjnl/qzaf040 (PMC12282763; doi:10.1093/gpbjnl/qzaf040)
Supplement: qzaf040_Supplementary_Data [file qzaf040_supplementary_data.zip › Table S4.docx]

**Table S4** **Features used to build ML variant classifier**

| **Category** | **Feature** | **Short name** | **Description** |
| --- | --- | --- | --- |
| Variant quality | 1 | FS | Phred-scaled p-value from Fisher's exact test of strand bias |
|  | 2 | GC | GC content within $\pm20\mathrm{bp}$ of a given variant position |
|  | 3 | HRun | Largest contiguous homopolymer run of variant allele in either direction (numeric) |
|  | 4 | MQ | Average mapping quality of variant-carrying reads |
|  | 5 | MQ0 | Number of variant-carrying reads with mapping quality of 0 |
|  | 6 | NBase | Percent of unknown (N) bases at the variant position in the pileup |
|  | 7 | STR | Variant is part of a short tandem repeat (0-no, 1-yes) |
|  | 8 | DP | Read depth at a given variant position |
|  | 9 | DPref | Read depth supporting the reference allele |
|  | 10 | DPalt | Read depth supporting the alternative allele |
|  | 11 | VAF | Variant allele frequency |
| Variant caller | 12 | Mutect | Mutect2 calling status (0-no, 1-yes) |
|  | 13 | VarDict | VarDict calling status (0-no, 1-yes) |
|  | 14 | VarTracker | VarTracker calling status (0-no, 1-yes) |
|  | 15 | MetaCaller | MetaCaller calling status (1-by one caller, 2-by two callers, and 3-by all three callers) |
| Mutation signature | 16 | MutSig | Mutational signature (0-C->T, 1-others) |
| Genomic context | 17 | Mscore | Mappability score of the variant position (range: 0–1) |
|  | 18 | Repeat | Repeat masked region (0-no, 1-yes) |
|  | 19 | LCR | Low complexity region (0-no, 1-yes) |
|  | 20 | CpG | CpG island (0-no, 1-yes) |
|  | 21 | SD | Segmental duplication region (0-no, 1-yes) |
| Population data | 22 | gnomAD.MAF | Overall MAF in gnomAD |
|  | 23 | 1000G.MAF | Overall MAF in 1000 Genomes Project |
|  | 24 | TOPMed.MAF | Overall MAF in TOPMed (NHLBI Trans-Omics for Precision Medicine) |
|  | 25 | rsID | Reference SNP in dbSNP (0-no match, 1-with match) |
|  | 26 | COSMIC.FREQ | Number of samples carrying the variant in the COSMIC database |

*Note*: Category includes variant quality (features 1–11), variant caller (features 12–15), mutation signature (feature 16), genomic context (features 17–21), and population data (features 22–26). Categorical variables include features 7, 12–16, 18–21, and 25. Feature 1 (“FS”) and feature 5 (“MQ0”) are not applicable to INDELs. For mappability score, only positions with mappability score $\leq$0.5 were kept (i.e., subsequences that occur at least twice in the genome); mappability score was from <http://hgdownload.cse.ucsc.edu/gbdb/hg19/bbi/wgEncodeCrgMapabilityAlign100mer.bw>. Repeats data were from https://genome.ucsc.edu/cgi-bin/hgTables (specify assembly:hg19, group:Repeats, and track:RepeatMasker). Low complex regions (LCR) were from <https://github.com/lh3/varcmp/raw/master/scripts/LCR-hs37d5.bed.gz>. CpG islands were from https://genome.ucsc.edu/cgi-bin/hgTables (specify assembly:hg19, group:Regulation, and track:CpG Islands). Segment duplicate (SD) data were from <https://eeelegacy.gs.washington.edu/humanparalogy/build37/data/GRCh37GenomicSuperDup.tab>. gnomAD data were from http://gnomad.broadinstitute.org/downloads (select gnomAD v2.1.1, Build=GRCH37). 1000G data were from ftp.1000genomes.ebi.ac.uk/vol1/ftp/release/20130502 (select *.20130502.genotypes.vcf.gz files). TOPMed data were from <https://bravo.sph.umich.edu/freeze3a/hg19/>. SNP data (rsID) were from https://ftp.ncbi.nih.gov/snp/archive/ (select b152/). COSMIC data were from https://cancer.sanger.ac.uk/cosmic/download (select GRCh37, v95). 1000G, 1000 Genomes Project; COSMIC, catalogue of somatic mutations in cancer; MAF, minor allele frequency; TOPMed, Trans-Omics for Precision Medicine.
