# Supplementary material for: UNISOM: Unified Somatic Calling and Machine Learning-based Classification Enhance the Discovery of CHIP
Source: Genomics Proteomics Bioinformatics. 2025 Apr 29;23(2):qzaf040. doi: 10.1093/gpbjnl/qzaf040 (PMC12282763; doi:10.1093/gpbjnl/qzaf040)
Supplement: qzaf040_Supplementary_Data [file qzaf040_supplementary_data.zip › Table S5.docx]

**Table S5 Performance metrics of neural network in CHIP prediction**

| **Type** | **Recall (%)** | **Precision (%)** | **Accuracy (%)** |
| --- | --- | --- | --- |
| SNV | 64.63 | 86.78 | 82.05 |
| INDEL | 90.26 | 91.07 | 87.92 |

*Note*: Recall, precision, and accuracy are estimated using formula (2), (1), and (5), respectively. Neural network was tested separately on SNV and INDEL, using WGS data from batch 2 simulation with CHIP-specific VAFs (Table S2).
