# Supplementary figures and images for: UNISOM: Unified Somatic Calling and Machine Learning-based Classification Enhance the Discovery of CHIP
Source: Genomics Proteomics Bioinformatics. 2025 Apr 29;23(2):qzaf040. doi: 10.1093/gpbjnl/qzaf040 (PMC12282763; doi:10.1093/gpbjnl/qzaf040)

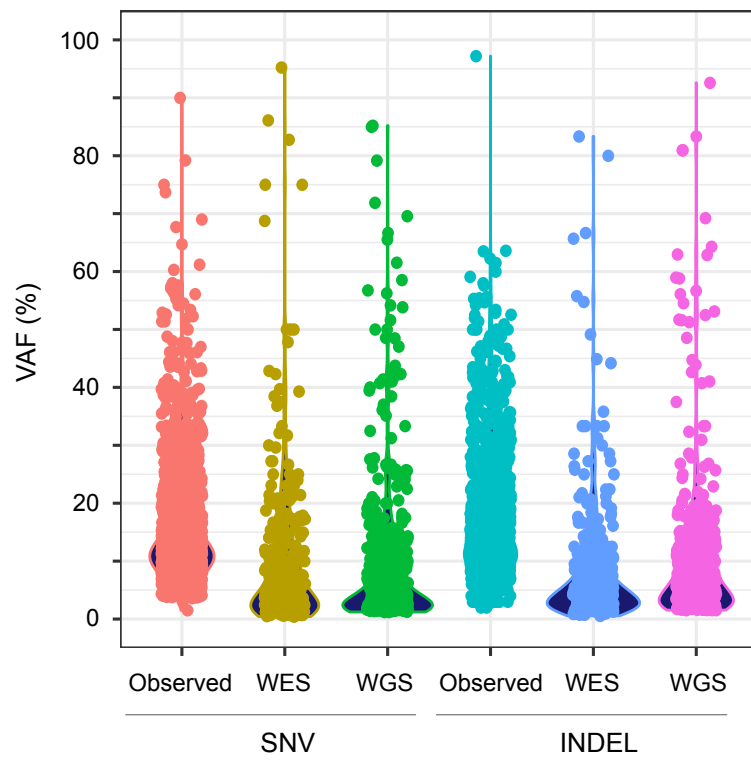

Supplement: qzaf040_Supplementary_Data [file qzaf040_supplementary_data.zip › Figure S2.pdf]

**A**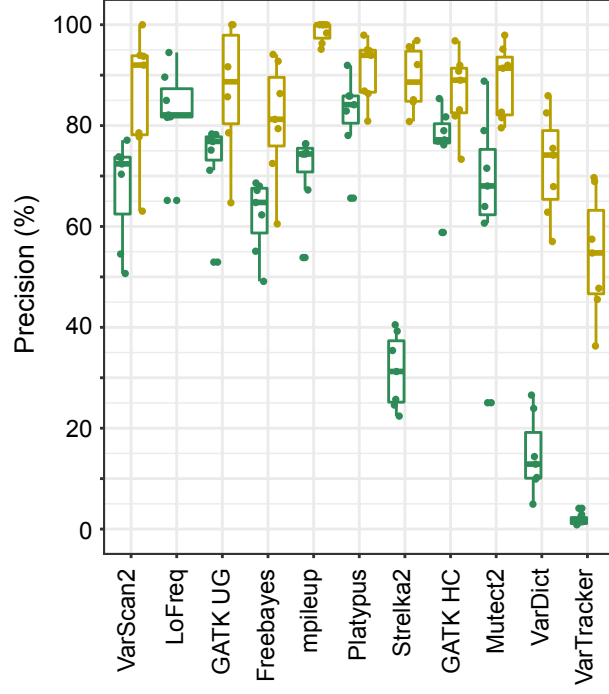**B**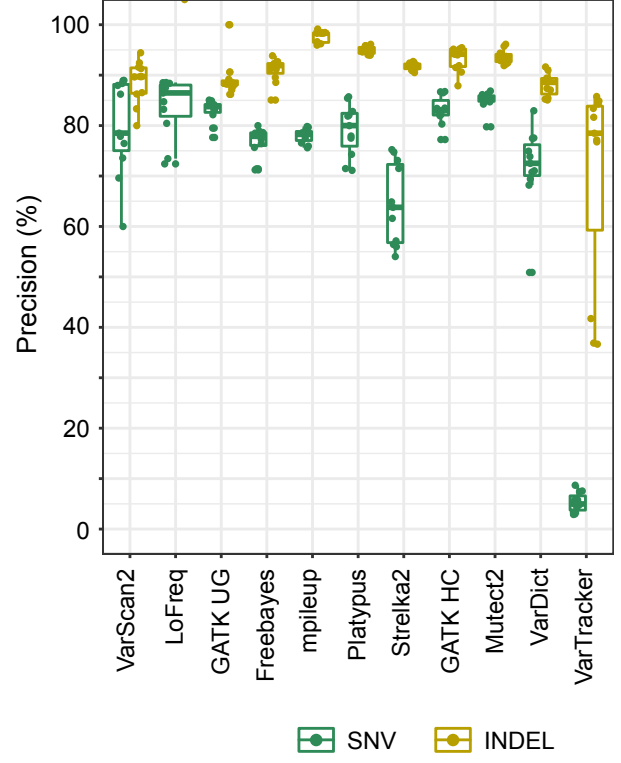

Supplement: qzaf040_Supplementary_Data [file qzaf040_supplementary_data.zip › Figure S4.pdf]

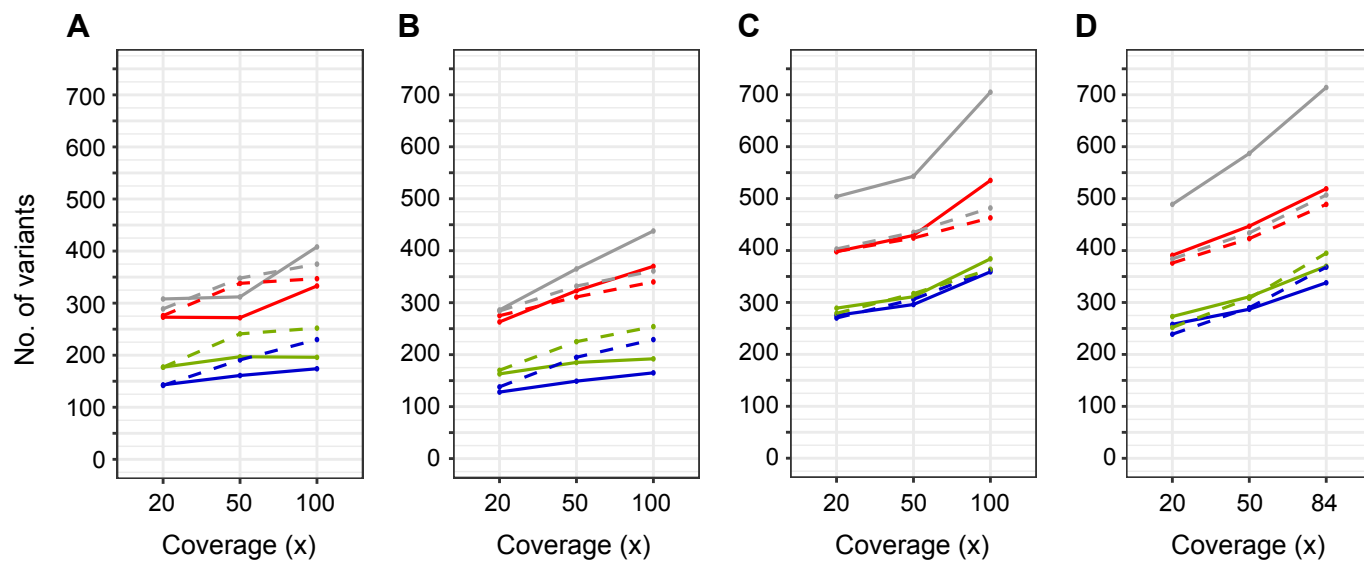

|              | SNV | INDEL   |
|--------------|-----|---------|
| No. spike-in | —●— | - -●- - |
| Mutect2      | —●— | - -●- - |
| VarDict      | —●— | - -●- - |
| VarTracker   | —●— | - -●- - |

Supplement: qzaf040_Supplementary_Data [file qzaf040_supplementary_data.zip › Figure S5.pdf]

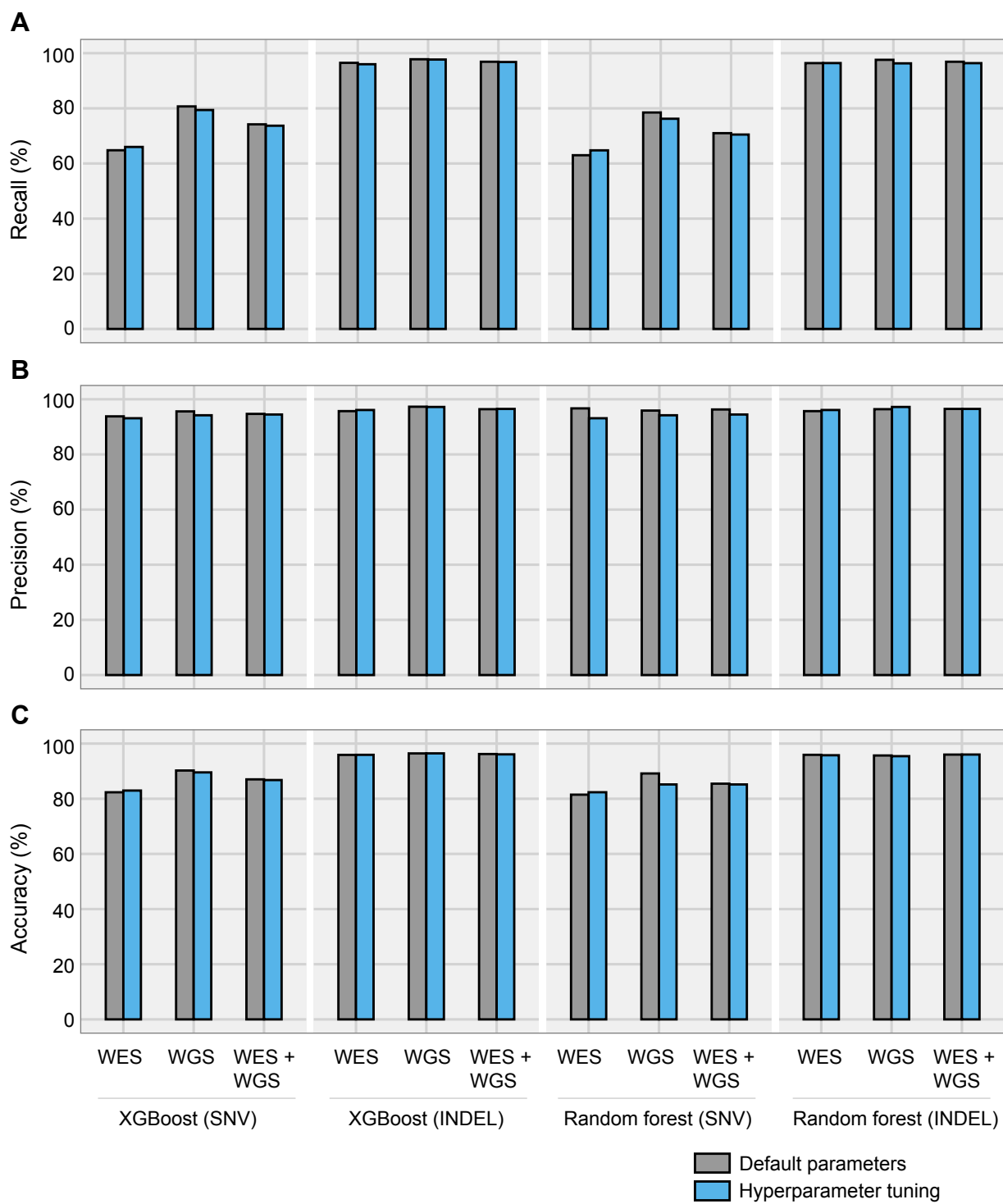

Supplement: qzaf040_Supplementary_Data [file qzaf040_supplementary_data.zip › Figure S6.pdf]

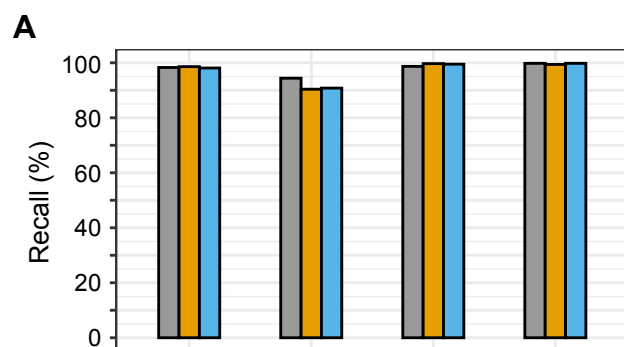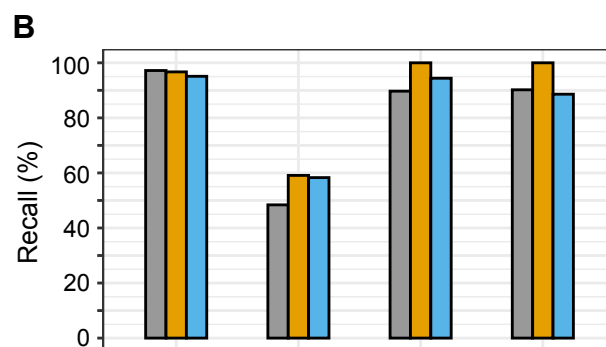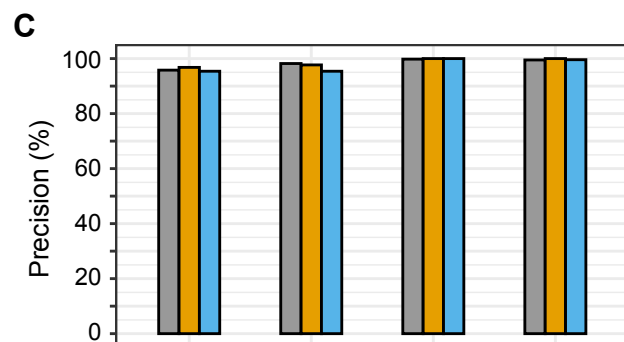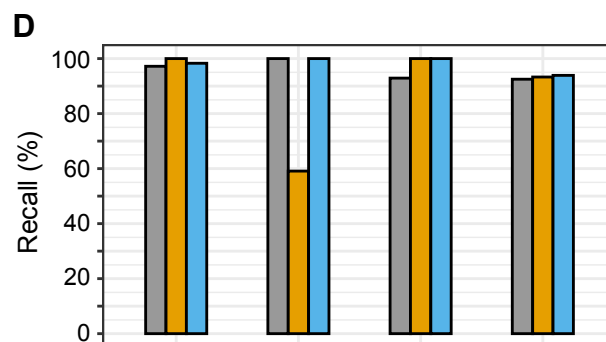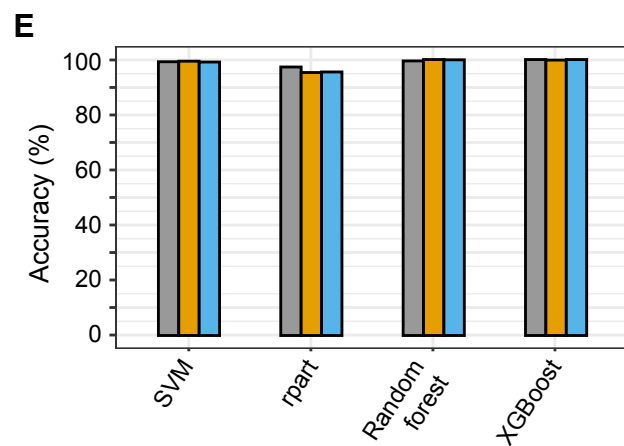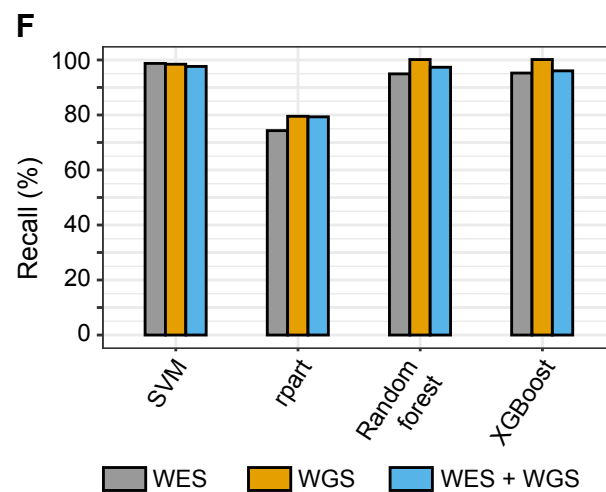

Supplement: qzaf040_Supplementary_Data [file qzaf040_supplementary_data.zip › Figure S7.pdf]

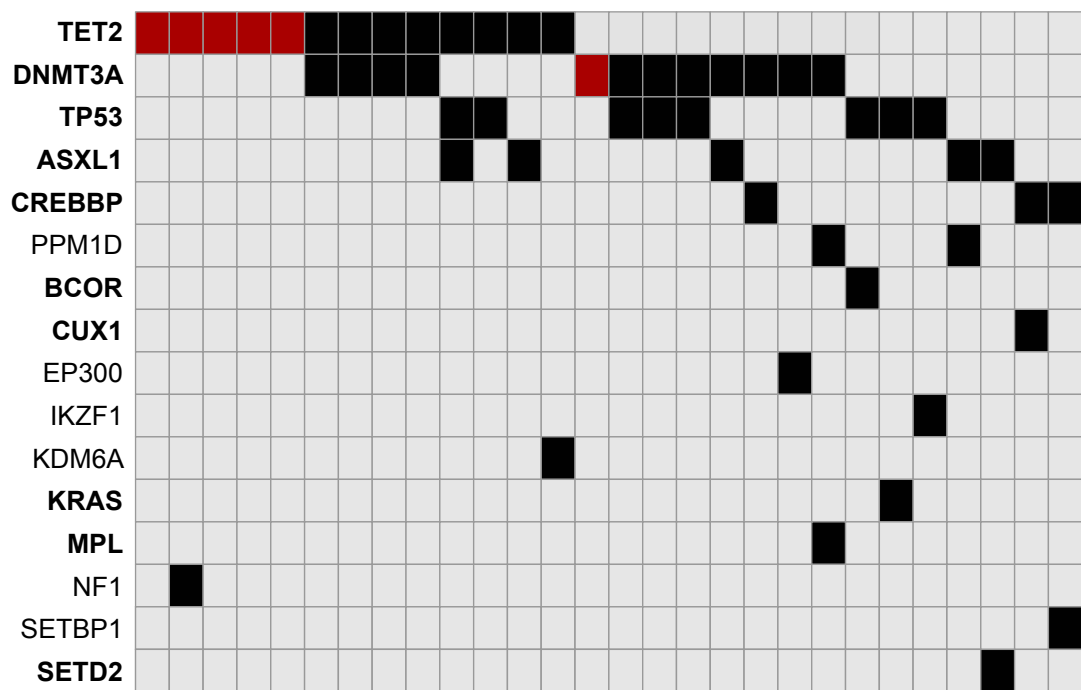

two mutations    one mutation    no mutation

Supplement: qzaf040_Supplementary_Data [file qzaf040_supplementary_data.zip › Figure S8.pdf]
